# Long non-coding RNAs as key orchestrators of the tumor microenvironment in lung cancer

**DOI:** 10.3389/fimmu.2025.1716180

**Published:** 2025-12-02

**Authors:** Xuechao Li, Yifei Ren, Hanming Hao, Yi Jin, Baoer Chen, Kai Zhao, Yishuo Ji, Guanyu Chen, Zhenglin He

**Affiliations:** 1 Norman Bethune College of Medicine, Jilin University, Changchun, China; 2 School and Hospital of Stomatology, Jilin University, Changchun, China; 3 College of Basic Medical Sciences, Jilin University, Changchun, China; 4 School of Cellular and Molecular Medicine, University of Bristol, Bristol, United Kingdom; 5 Department of Clinical Nutrition, Xinhua Hospital Affiliated to Shanghai Jiao Tong University School of Medicine, Shanghai, China; 6 School of Public Health, Shanghai Jiao Tong University School of Medicine, Shanghai, China; 7 Department of Immunobiology, Yale University School of Medicine, New Haven, CT, United States; 8 Center of Molecular and Cellular Oncology, Yale Cancer Center, Yale University, New Haven, CT, United States; 9 Department of Respiratory Medicine, The First Hospital of Jilin University, Changchun, China; 10 Department of Experimental Orofacial Medicine, University of Marburg, Marburg, Germany; 11 Department of Biobank, China-Japan Union Hospital of Jilin University, Changchun, China

**Keywords:** long non-coding RNAs (lncRNAs), lung cancer, tumor microenvironment (TME), biomarkers, therapy resistance

## Abstract

Long non-coding RNAs (lncRNAs) are emerging as master regulators of the lung cancer tumor microenvironment (TME), where they reprogram immune cell functions, cytokine networks, and checkpoint signaling to create an immunosuppressive and therapy-resistant landscape. This review offers a comprehensive analysis of how lncRNAs mediate the interplay between tumor cells and immune components, underscoring their context-dependent roles as both oncogenic drivers and microenvironmental suppressors. It also highlights their clinical utility as liquid-biopsy biomarkers and their central role in conferring resistance to chemo- and radio-therapy. This review synthesizes current knowledge on the interplay between lncRNAs and the TME, highlighting the targeting of specific lncRNAs as a novel therapeutic strategy for precision lung cancer therapy.

## 1 Introduction

Lung cancer remains the leading cause of cancer-related mortality worldwide, characterized by frequent late-stage diagnosis and a persistently poor prognosis for advanced disease (1–4). A major driver of this aggressiveness is the highly complex and dynamic tumor microenvironment (TME), which plays a fundamental role in promoting tumor progression, metastasis, and therapeutic resistance (5). Despite advances in surgery, targeted therapy, and radiation, survival for advanced disease remains unsatisfactory, largely due to immune evasion and treatment resistance (6).

While immune checkpoint inhibitors (ICIs) and adoptive cell therapies like chimeric antigen receptor T-cell immunotherapy (CAR-T) have revolutionized lung cancer treatment, their efficacy remains limited and heterogeneous (7). Objective response rates are often modest, and acquired resistance is common (8, 9). A fundamental barrier to the success of these immunotherapies is the profoundly immunosuppressive nature of the TME (10, 11). The TME comprises heterogeneous cellular components including tumor cells, diverse immune cells, cancer-associated fibroblasts (CAFs), endothelial cells, and extracellular matrix that interact through complex signaling networks to create an immunosuppressive milieu promoting tumor growth and metastasis (12, 13). Immune cells within the TME display functional plasticity and often support tumor progression through cytokine dysregulation, checkpoint expression, metabolic competition, and effector cell suppression (12). Therefore, a comprehensive understanding and therapeutic targeting of the immunosuppressive TME is essential to overcome resistance and enhance the efficacy of therapeutic strategies (14).

**Figure 1:**
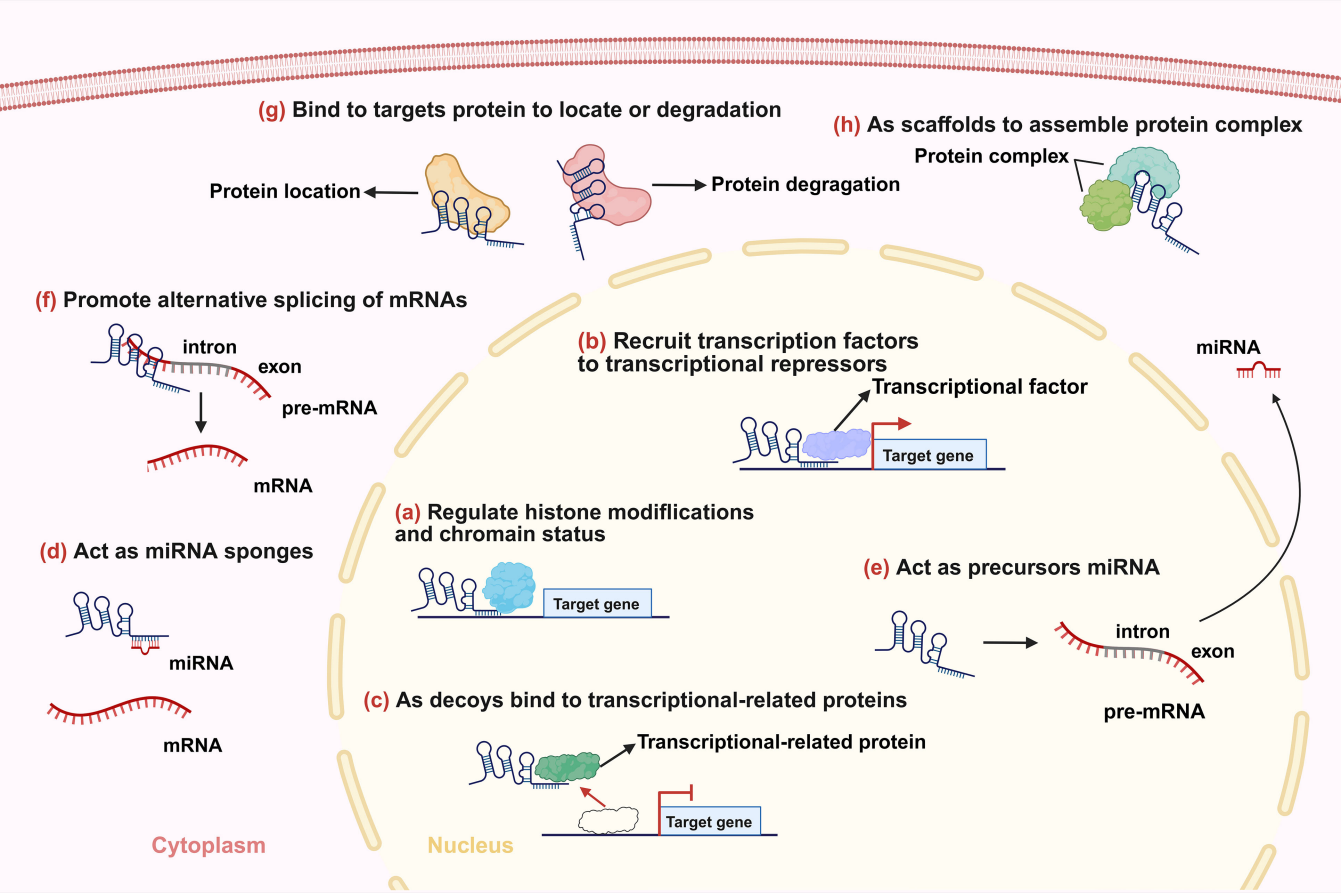
Main mechanisms of lncRNAs. **(a)** lncRNAs regulate histone modifications and/or chromatin status. **(b)** lncRNAs recruit transcription factors or transcriptional repressors to the promoters of their target genes to regulate transcription. **(c)** lncRNAs bind to transcriptional-related proteins as decoys preventing them from binding to their DNA targets. **(d)** lncRNAs function as miRNA sponges. **(e)** lncRNAs act as precursors of some small RNAs. **(f)** lncRNAs promote alternative splicing of mRNAs. **(g)** lncRNAs influence localization and degradation of targets proteins. **(h)** lncRNAs act as scaffolds to promote the formation of protein complexes.

**Figure 2:**
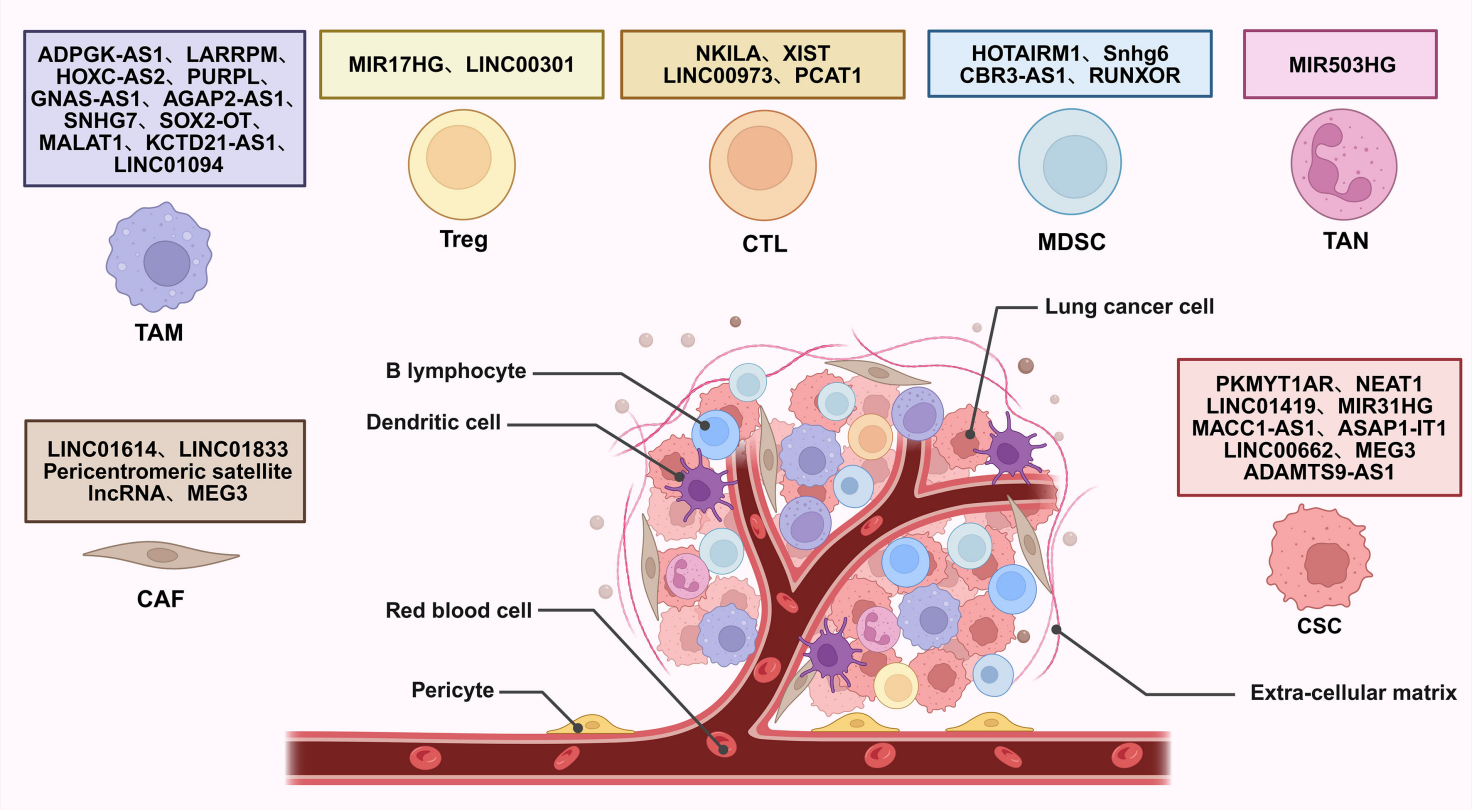
Interaction of lncRNAs with TME regulating lung cancer development. CAF, cancer-associated fibroblast; TAM, tumor-associated macrophage; Treg, regulatory T cell; CTL, cytotoxic T lymphocyte; MDSC, myeloid-derived suppressor cell; TAN, tumor-associated neutrophil; CSC, cancer stem cell.

Long non-coding RNAs (lncRNAs) are generally defined as RNA transcripts longer than 200 nucleotides with limited or no protein-coding potential (15). They have emerged as pivotal regulators of gene expression through diverse mechanisms, acting at transcriptional, post-transcriptional, and epigenetic levels (**Figure 1**) (15, 16). They modulate fundamental processes including proliferation, apoptosis, differentiation, metastasis, metabolic reprogramming, and crucially, immune responses (17). Within the complex milieu of the lung cancer TME, lncRNAs emerge as critical mediators of intercellular communication and microenvironmental reprogramming (**Figure 2**) (18). By directly governing immune cell function and polarization, influencing the expression of immune-related genes and checkpoints, and modulating tumor-stromal interactions, lncRNAs collectively sculpt an immunosuppressive niche that promotes tumor growth, metastasis, and therapy resistance (19).

**Figure 3:**
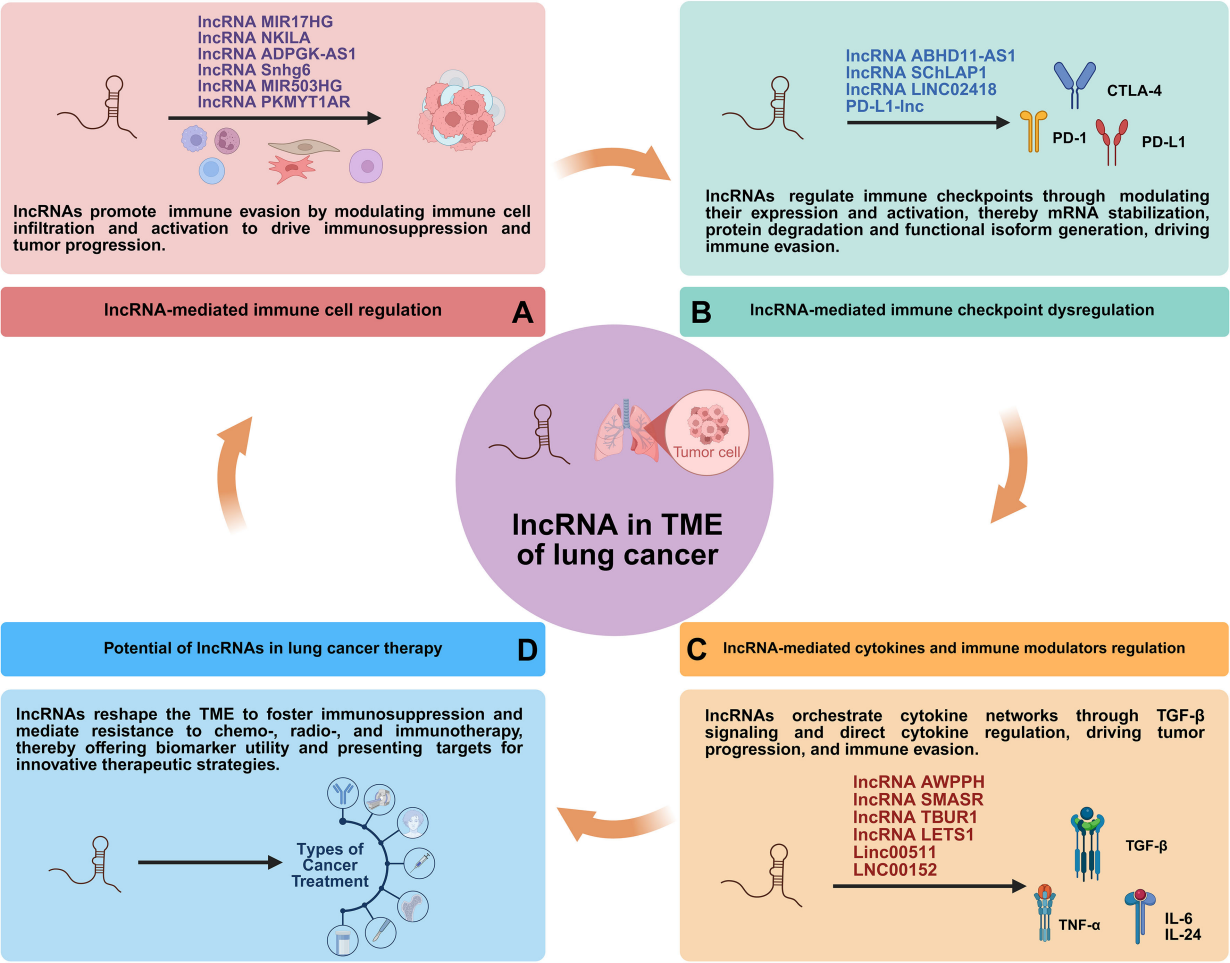
Schematic representations of roles of lncRNA in lung cancer TME.

While numerous individual lncRNAs have been characterized, a unifying perspective that integrates their roles across the entire immunosuppressive landscape is lacking. To bridge this knowledge gap, this review provides a comprehensive and contextualized analysis of lncRNAs as central orchestrators of the lung cancer TME. We focus on their coordinated regulation of immune components, stromal cells, and signaling networks. Furthermore, we critically evaluate their emerging roles as liquid biopsy biomarkers and therapeutic targets, thereby mapping a path from mechanistic understanding to clinical translation. Ultimately, this synthesis aims to highlight novel therapeutic targets and outline combinatorial approaches for overcoming resistance in lung cancer (**Figure 3**).

## 2 TME in lung cancer

TME is a pivotal determinant of cancer progression and therapeutic outcome, such as in lung cancer (20). It consists of malignant cells, diverse immune populations, stromal components, and molecular factors such as immune checkpoints and cytokines, all engaged in dynamic crosstalk. These interactions collectively promote immunosuppression, metastasis, and therapeutic resistance (21, 22). In lung cancer, the TME evolves with disease progression, adapting to therapeutic pressures and shaping clinical outcomes (22).

The cellular composition of the lung cancer TME includes a diverse array of immune and stromal cells. Cytotoxic T lymphocytes (CTLs) and regulatory T cells (Tregs) play opposing roles. CTLs mediate antitumor responses, while Tregs suppress effector immunity through cytokines like interleukin 10 (IL-10) and transforming growth factor beta (TGF-β) (23, 24). Myeloid-derived suppressor cells (MDSCs) inhibit T-cell function via arginase-1 (Arg-1) and reactive oxygen species (ROS) (24). Tumor-associated macrophages (TAMs), particularly M2-polarized subsets, facilitate angiogenesis and tissue remodeling through vascular endothelial growth factor (VEGF) and chemokine ligand 2 (CCL2) secretion (25). Tumor-associated neutrophils (TANs) promote metastasis via neutrophil extracellular traps (NETs), inducing epithelial-mesenchymal transition (EMT) (26). Additionally, cancer-associated fibroblasts (CAFs) and cancer stem cells (CSCs) contribute to extracellular matrix remodeling, growth factor release, and drug resistance, further reinforcing the immunosuppressive niche in lung cancer (27, 28).

Immune checkpoints constitute pivotal immunosuppressive mechanisms exploited by tumors to evade immunity within the TME. Programmed cell death ligand 1 (PD-L1) overexpression on tumor/stromal cells engages programmed death receptor 1 (PD-1) to exhaust T cells, while cytotoxic T lymphocyte associate protein-4 (CTLA-4) competitively blocks CD28 co-stimulation (29). Critically, tumor-intrinsic drivers synergistically reinforce immune evasion. These integrated mechanisms establish dominant resistance to cytotoxic lymphocytes, with checkpoint blockade demonstrating transformative efficacy in lung cancer (30).

Cytokines further shape the immune contexture of lung cancer. TGF-β promotes EMT and metastatic progression in lung cancer, while IL-6 activates signal transducer and activator of transcription 3 (STAT3) signaling to support tumor cell survival and proliferation (31, 32). Tumor necrosis factor alpha (TNF-α) contributes to cancer-related inflammation and angiogenesis through nuclear factor kappa B (NF-κB) activation (33). These cytokines collectively enhance the recruitment and function of immunosuppressive cells while inhibiting effector immune responses, thereby sustaining a chronic inflammatory state that favors lung cancer progression.

The roles of lncRNAs in the differentiation and function of various immune cells, including T cells, TAMs, MDSCs and TANs, as well as other tumor associated cells are increasingly well understood (34). Critically, lncRNAs interface with cytokine signaling and immune checkpoint pathways, thereby serving as both biomarkers and actionable targets for immunotherapy potentiation of lung cancer.

## 3 lncRNA regulation of the TME in lung cancer

lncRNAs have been shown to play a crucial role in modulating TME in lung cancer through various mechanisms. They participate in the regulation of immune cell functions, cytokine production, and checkpoint expression, thereby influencing antitumor immunity and therapeutic efficacy. This section discusses the interplay between lncRNAs and different immune components in the context of lung cancer (**Table 1**).

**Table 1:** lncRNAs orchestrating TME of lung cancer.

lncRNAs	Cancer types	Regulation	Effects	Targets	Mechanisms	Ref.
MIR17HG	NSCLC	Downregulated	Tregs	miR-17-5p/RUNX3	Downregulates miR-17-5p to inhibit RUNX3, promoting Treg expansion and immunosuppressive cytokine production.	(35)
LINC00301	NSCLC	Upregulated	Tregs	EZH2/EAF2/HIF1α	Recruits EZH2 to repress EAF2 and sponges miR-1276, amplifying HIF1α expression.	(36)
NKILA	Lung cancer	Upregulated	CTLs	NF-κB/IκB complex	Binds NF-κB/IκB complex, inhibits NF-κB signaling, sensitizes CTLs to activation-induced cell death.	(37)
XIST	Lung cancer	Upregulated	CTLs	miR-34a-5p/PD-L1	Downregulates miR-34a-5p to upregulate PD-L1, inhibiting CD8^+^ T cell cytotoxicity.	(38)
LINC00973	Lung cancer	Upregulated	CTLs	miR-216b/miR-150	Sponges miR-216b/miR-150 to upregulate CD55/CD59, inhibiting complement lysis and CTL activation.	(39)
PCAT1	NSCLC	Upregulated	CTLs	SOX2/cGAS	Activates SOX2 to repress cGAS/STING signaling, impairing DC maturation and CD8^+^ T cell priming.	(40)
ADPGK-AS1	Lung cancer	Upregulated	TAMs	MRPL35	Binds MRPL35, enhances mitochondrial metabolism and fission, driving M2 polarization.	(41)
LARRPM	LUAD	Downregulated	TAMs	TET1/LINC00240/CSF1	Recruits TET1 to demethylate LINC00240 promoter and reduces TET1 binding to the CSF1 promoter, increasing its methylation, restricting M2 polarization	(42)
HOXC-AS2	NSCLC	Upregulated	TAMs	HOXC13/STAT1	Silences STAT1 signaling, promoting M2 polarization.	(43)
PURPL	Lung cancer	Upregulated	TAMs	RBM4/xCT	Upregulates xCT-mediated programs, promoting M2 polarization.	(44)
GNAS-AS1	NSCLC	Upregulated	TAMs	miR-4319/NECAB3	Sponges miR-4319 to upregulate NECAB3, promoting M2 polarization.	(45)
AGAP2-AS1	Lung cancer	Upregulated	TAMs	miR-296/NOTCH2	Downregulates miR-296 to upregulate NOTCH2, enhancing radioresistance.	(46)
SNHG7	LUAD	Upregulated	TAMs	ATG5/ATG12/PTEN	Induces autophagy and activates PI3K/AKT pathway, driving M2 polarization.	(47)
SOX2-OT	NSCLC	Upregulated	TAMs	miR-627-3p/Smad2/Smad3	Promotes macrophages M2 polarization via promoting Smads by sponging miR-627-3p.	(48)
MALAT1	LUAD	Upregulated	TAMs	CCL2	Recruits pro-tumorigenic macrophages by upregulating CCL2 secretion through chromatin remodeling.	(49)
KCTD21-AS1	NSCLC	Upregulated	TAMs	TIPRL/CD47	Upregulates CD47 and promotes autophagy, impairing macrophage phagocytosis.	(50)
LINC01094	LUAD	Upregulated	TAMs	SPI1/CCL7	Promotes SPI1-dependent CCL7 transcription to recruit M2 macrophages.	(51)
HOTAIRM1	Lung cancer	Downregulated	MDSCs	HOXA1	Enhances HOXA1 expression, which curtails MDSC immunosuppressive function.	(52)
Snhg6	Lung cancer	Upregulated	MDSCs	EZH2	Promotes EZH2 degradation, facilitating M-MDSC differentiation.	(53)
CBR3-AS1	NSCLC	Upregulated	MDSCs	miR-409-3p/CXCL1	Sponges miR-409-3p to upregulate CXCL1, recruiting MDSCs and inhibiting T cells.	(54)
RUNXOR	Lung cancer	Upregulated	MDSCs	RUNX1	Represses RUNX1, upregulating Arg-1 to foster immune escape.	(55)
MIR503HG	NSCLC	Downregulated	TANs	C/EBPβ/NLRP3	Downregulated by NETs via methylation. Enables C/EBPβ accumulation and NLRP3 inflammasome activation, driving metastasis.	(56)
PKMYT1AR	NSCLC	Upregulated	CSCs	miR-485-5p/PKMYT1	Sponges miR-485-5p to stabilize PKMYT1, inhibiting β-catenin degradation and driving CSC maintenance.	(57)
NEAT1	NSCLC	Upregulated	CSCs	hsa-mir-98-5p/CTR1	Inhibits copper transporter 1, potentiating EMT cascades for CSC maintenance.	(58)
LINC01419	LUAD	Upregulated	CSCs	EZH2/FBP1	Recruits EZH2 to epigenetically silence FBP1, activating Hippo signaling and conferring chemoresistance in CD44+ cells	(59)
MIR31HG	NSCLC	Upregulated	CSCs	GLI2/WDR5	Recruits WDR5/MLL3/P300 complex to deposit H3K4me1/H3K27ac at GLI2 promoter, augmenting its transcription and conferring CSC-like properties.	(60)
MACC1-AS1	NSCLC	Upregulated	CSCs	UPF1/LATS1/LATS2	Interacts with UPF1 to destabilize LATS1/2 mRNA, inhibiting the Hippo pathway.	(61)
ASAP1-IT1	NSCLC	Upregulated	CSCs	miR-509-3p/YAP1	Sponges miR-509-3p to derepress YAP1, enhancing cancer stemness.	(62)
LINC00662	Lung cancer	Upregulated	CSCs	Lin28/miR-16-5p	Interacts with Lin28 to promote invasion and CSC-like phenotypes.	(63)
MEG3	Lung cancer	Downregulated	CSCs	miR-650/SLC34A2	Sponges miR-650 to derepress SLC34A2, suppressing migration and invasion of lung CSCs.	(64)
ADAMTS9-AS1	LUAD	Downregulated	CSCs	miR-5009-3p/NPNT	Upregulates miR-5009-3p, which targets nephronectin, thereby suppressing cell stemness.	(65)
LINC01614	LUAD	Upregulated	CAFs	Annexin A2/p65	Binds Annexin A2/p65 to activate NF-κB, upregulating SLC38A2/SLC7A5 to enhance glutamine uptake in tumor cells.	(66)
Pericentromeric satellite lncRNA	Lung cancer	Upregulated	CAFs	inflammatory genes	Activated by stromal stimuli (TGF-β, IL-1α, etc.). Essential for maintaining inflammatory CAF phenotype and cellular senescence.	(67)
LINC01833	NSCLC	Upregulated	CAFs	miR-335-5p/VAPA	Sponges miR-335-5p to upregulate VAPA, promoting proliferation, migration, and invasion	(68)
MEG3	SCLC	Upregulated	CAFs	miR-15a-5p/CCNE1	Binds miR-15a-5p to derepress CCNE1, driving cisplatin chemoresistance.	(69)
ABHD11-AS1	Lung cancer	Upregulated	PD-L1	SART3/USP15/TRAF3	Activates non-canonical NF-κB/IL-6/STAT3 pathway to upregulate PD-L1 transcription.	(70)
SChLAP1	NSCLC	Upregulated	PD-L1	AUF1/PD-L1	Recruits AUF1 to stabilize PD-L1 mRNA, increasing PD-L1 protein levels.	(71)
LINC02418	NSCLC	Downregulated	PD-L1	TRIM21/PD-L1	Scaffolds TRIM21 to induce PD-L1 ubiquitination and degradation.	(72)
PD-L1-lnc	LUAD	Upregulated	PD-L1	c-Myc	Binds and stabilizes c-Myc, driving immune-independent tumor progression.	(73)
AWPPH	NSCLC	Upregulated	TGF-β	TGF-β1	Directly elevates TGF-β1 production, creating a pro-metastatic autocrine loop.	(74)
SMASR	Lung cancer	Downregulated	TGF-β	TGFBR1/Smad2/3	Binds unphosphorylated Smad2/3 to block TGF-β receptor activation and inhibits TGFBR1 transcription.	(75)
TBUR1	LUAD	Upregulated	TGF-β	hnRNPC/GRB2 mRNA	Binds hnRNPC to stabilize GRB2 mRNA in an m6A-dependent manner, potentiating ERK/MAPK signaling for EMT.	(76)
LINC01977	LUAD	Upregulated	TGF-β	Smad3/ZEB1	Hijacks super-enhancers, facilitates Smad3 nuclear import and recruitment to the ZEB1 promoter.	(77)
TBILA	NSCLC	Upregulated	TGF-β	HGAL/S100A7	Binds nuclear Smads to enhance RhoA activation and activates the S100A7-JAB1 pro-survival pathway.	(78)
LETS1	Lung cancer	Upregulated	TGF-β	NR4A1/SMAD7	Induces NR4A1 to target SMAD7 for degradation, stabilizing TGF-β receptor and enhancing signaling.	(79)
HCP5	LUAD	Upregulated	TGF-β	miR-203/SMAD3	Sponges miR-203 to derepress SNAI1/SNAI2, initiating EMT.	(80)
ELIT-1	Lung cancer	Upregulated	TGF-β	Smad3/Snail	Binds Smad3 as a cofactor to facilitate its recruitment to target gene promoters.	(81)
LINC00511	Lung cancer	Upregulated	TGF-β	miR-183-5p/ZEB2	Sponges miR-183-5p to derepress ZEB2, activating EMT programs.	(82)
XIST	NSCLC	Upregulated	TGF-β	miR-367/141	Sponges miR-367/141 to derepress ZEB2, driving EMT.	(83)
LEISA	LUAD	Upregulated	IL-6	STAT3/IL-6	Recruits STAT3 to the IL-6 promoter, augmenting IL-6 secretion and STAT3 signaling.	(84)
LINC00152	LUAD	Upregulated	IL-24	EZH2/IL24	Represses the expression of the pro-apoptotic cytokine IL24 by recruiting EZH2 to its promoter.	(85)
THRIL	NSCLC	Downregulated	TNF-α	TNF-α/HuR	Forms a regulatory circuit with TNF-α. Stabilized by HuR but degraded via m6A/YTHDF2 pathway.	(86)

NSCLC, Non-small cell lung cancer; LUAD, Lung adenocarcinoma; Treg, Regulatory T cell; EZH2, Enhancer of zeste homolog 2; RUNX3, Runt-related transcription factor 3; EAF2, ELL associated factor 2; HIF1α, Hypoxia-inducible factor 1 alpha; CTLs, Cytotoxic T lymphocytes; NF-κB, Nuclear factor kappa B; IκB, Inhibitor of kappa B; AICD, Activation-induced cell death; PD-L1, Programmed cell death 1 ligand 1; CD55, Cluster of Differentiation 55; CD59, Cluster of Differentiation 59; SOX2, SRY-Box Transcription Factor 2; cGAS, cyclic GMP-AMP synthase; STING, Stimulator of interferon genes; TAMs, Tumor-associated macrophages; MRPL35, Mitochondrial ribosomal protein L35; M2, M2 macrophage; TET1, Ten-eleven translocation 1; CSF1, Colony stimulating factor 1; STAT1, Signal transducer and activator of transcription 1; RBM4, RNA-binding motif protein 4; xCT, Cystine/glutamate antiporter; NECAB3, N-terminal EF-hand calcium binding protein 3; NOTCH2, Neurogenic locus notch homolog protein 2; ATG5, Autophagy related 5 Gene; ATG12, Autophagy related 12 homolog; PTEN, Phosphatase and tensin homolog deleted on chromosome ten; PI3K/AKT, Phosphoinositide 3-kinase/protein kinase B; Smads, Mothers against decapentaplegic homolog; CCL2, C-C motif chemokine ligand 2; TIPRL, TOR signaling pathway regulator; CD47, Cluster of differentiation 47; SPI1, Spleen focus forming virus proviral integration oncogene; CCL7, C-C motif chemokine ligand 7; MDSCs, Myeloid-derived suppressor cells; HOXA1, Homeobox A1; CXCL1, C-X-C motif chemokine ligand 1; Arg-1, Arginase-1; TANs, Tumor-associated neutrophils; C/EBPβ, CCAAT/enhancer binding protein beta; NLRP3, NOD-like receptor protein 3; CSCs, Cancer stem cells; PKMYT1, Membrane-associated tyrosine- and threonine-specific cdc2-inhibitory kinase; CTR1, Copper Transporter 1; EMT, Epithelial-mesenchymal transition; FBP1, Fructose-1,6-bisphosphatase 1; WDR5, WD repeat-containing protein 5; MLL3, Mixed lineage leukemia 3; GLI2, GLI family zinc finger 2; UPF1, UPF1 regulator of nonsense mediated mRNA decay; LATS1/2, Large tumor suppressor kinase 1/2; YAP1, Yes-associated protein 1; Hippo, Hippo signaling pathway; Lin28, Lin-28 homolog A; SLC34A2, Solute carrier family 34 member 2; NPNT, Nephronectin; CAFs, Cancer-associated fibroblasts; Annexin A2, Annexin A2; p65, NF-κB p65 subunit; SLC38A2, Solute carrier family 38 member 2; SLC7A5, Solute carrier family 7 member 5; VAPA, VAMP associated protein A; CCNE1, Cyclin E1; USP15, Ubiquitin specific peptidase 15; TRAF3, TNF receptor-associated factor 3; AUF1, AU-rich element RNA binding protein 1; TRIM21, Tripartite motif containing 21; c-Myc, MYC proto-oncogene; TGF-β, Transforming growth factor beta; hnRNPC, Heterogeneous nuclear ribonucleoprotein C; GRB2, Growth factor receptor bound protein 2; ERK, Extracellular signal-regulated kinase; MAPK, Mitogen-activated protein kinase; ZEB1, Zinc finger E-box binding homeobox 1; HGAL, Human Germinal Center Associated Lymphoma; RhoA, Ras homolog family member A; S100A7, S100 calcium binding protein A7; JAB1, Jun activation domain binding protein 1; NR4A1, Nuclear receptor subfamily 4 group A member 1; SMAD7, SMAD family member 7; SNAI, Snail family transcriptional repressor; STAT3, Signal transducer and activator of transcription 3; IL-6, Interleukin 6; IL-24, Interleukin 24; TNF-α, Tumor necrosis factor alpha; HuR, Human antigen R; YTHDF2, YTH n6-methyladenosine RNA binding protein 2.

### 3.1 Regulation of immune cells by lncRNAs in lung cancer

#### 3.1.1 Adaptive immune cells

**Figure 4:**
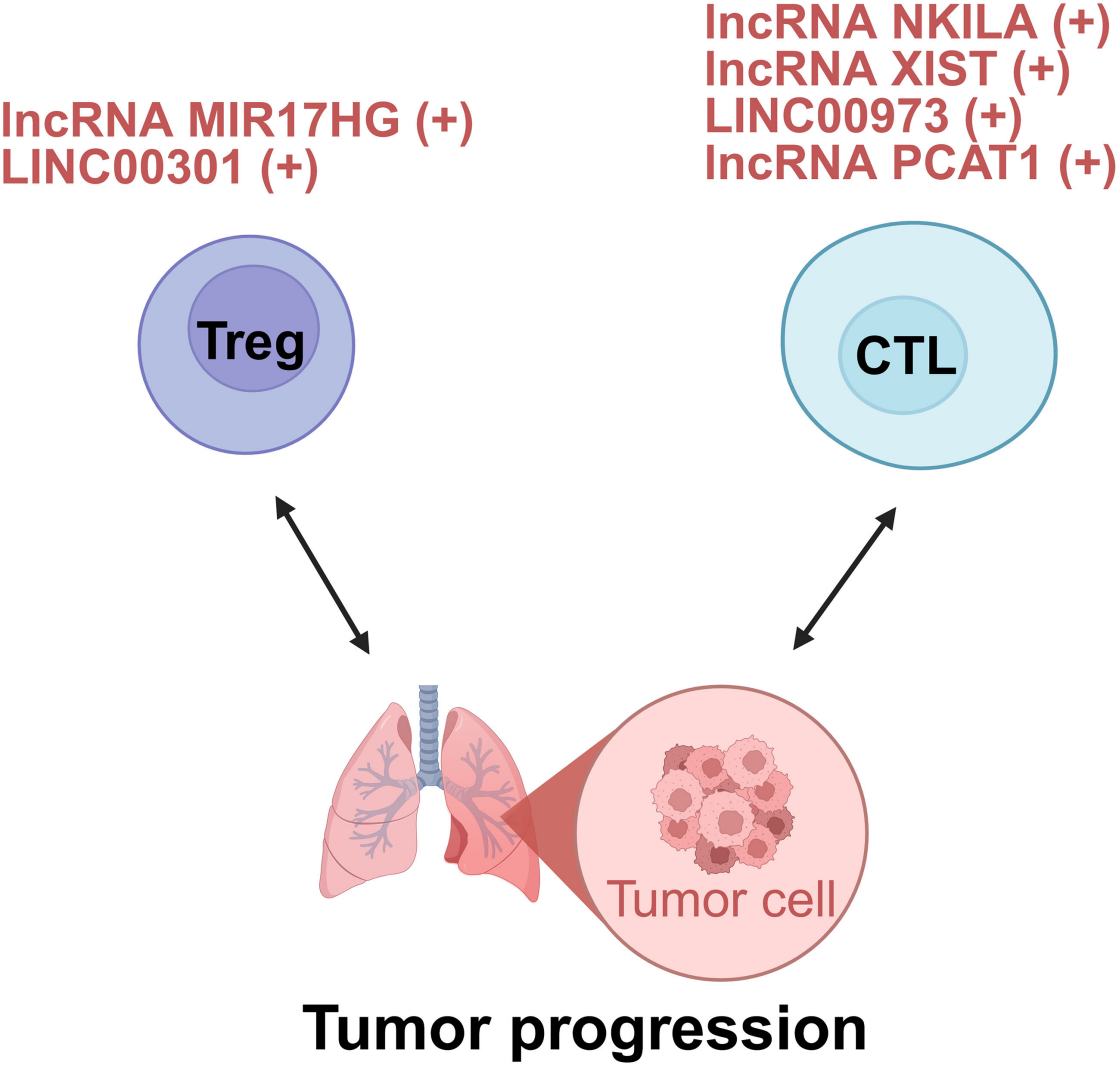
lncRNAs act as modulators between adaptive cells and tumor cells of lung cancer. +: promoting the tumor progression; -: inhibiting the tumor progression. Treg, regulatory T cell; CTL, cytotoxic T lymphocyte.

Adaptive immune cells, including B lymphocytes and T lymphocytes, mediate humoral and cellular immune responses. Current studies regarding lncRNAs in lung cancer and adaptive immunity have largely focused on T cells, especially Tregs and CTLs, due to limited evidence regarding B cell-related lncRNA mechanisms (**Figure 4**).

##### 3.1.1.1 Tregs

In the TME of lung cancer, Tregs inhibit the activity of effector T cells, limit the function of antigen-presenting cells, and secrete immunosuppressive cytokines like IL-10 and TGF-β. These actions collectively dampen the immune system’s ability to attack tumor cells, enabling tumors to evade immune surveillance and promoting their growth and progression. The presence of a large number of Tregs in tumor tissues is often associated with poor clinical prognosis (23). Emerging evidence indicates that lncRNAs critically regulate Treg differentiation, function, and immunosuppressive activity in lung cancer.

In the realm of tumor immunology, understanding how lncRNAs modulate Treg-mediated immune evasion has become a focal point. lncRNA miR-17-92a-1 cluster host gene (MIR17HG), upregulated in non-small cell lung cancer (NSCLC), promotes Treg-mediated immunosuppression. Mechanistically, it serves as a precursor for miR-17-5p, which directly targets and downregulates runt-related transcription factor 3 (RUNX3). Consequently, RUNX3 deficiency amplifies Treg expansion and enhances immunosuppressive cytokine production (VEGF-A, TGF-β, IL-10), while suppressing interferon-gamma (IFN-γ) secretion. Critically, MIR17HG knockdown *in vivo* reduces Treg infiltration and suppresses tumor growth, highlighting its role as a pro-tumorigenic lncRNA (35). However, whether altering RUNX3 expression could affect the functions of MIR17HG and miR-17-5p in the tumorigenicity or Treg-mediated immune escape of NSCLC cells should be further investigated (35).

Beyond their role in immune evasion, lncRNAs also play a key role in shaping the immune-suppressive TME through their regulation of Tregs. For instance, LINC00301, transcriptionally activated by forkhead box C1 (FOXC1) and overexpressed in advanced NSCLC, orchestrates immunosuppression via dual-pathway potentiation of hypoxia-inducible factor 1 alpha (HIF1α). In the nucleus, LINC00301 directly binds enhancer of zeste homolog 2 (EZH2), recruiting it to deposit repressive trimethylated histone H3 at lysine 27 (H3K27me3) marks on the ELL associated factor 2 (EAF2) promoter. Consequently, EAF2 deficiency destabilizes von Hippel–Lindau (VHL) protein, leading to hypoxia-inducible factor 1α (HIF1α) accumulation. In the cytoplasm, LINC00301 functions as a competitive endogenous RNA (ceRNA) to sponge miR-1276, thereby relieving miR-1276-mediated suppression of HIF1α mRNA and further amplifying HIF1α expression (36). Notably, this study demonstrated that the primary oncogenic driver of LINC00301 is its nuclear role in binding EZH2, while the contribution of its cytoplasmic ceRNA function appears minimal. Thus, the proposed ceRNA mechanism warrants further validation (36).

Indeed, the lncRNA-Treg axis is a central driver of immune evasion in lung cancer. Pinpointing the few lncRNAs that sit at the crossroads of this axis may provide the missing leverage to restore anti-tumor immunity.

##### 3.1.1.2 CTLs

CD8^+^ T cells kill tumors via cytotoxic granules, but the TME suppresses them through nutrient depletion and immunosuppressive cells (87). Recent studies reveal that lncRNAs disable these T cells in two ways: directly undermining their survival and effector signaling, and indirectly disrupting innate-immune priming and checkpoint control. Targeting these lncRNAs offers a route to reinvigorate CD8^+^ T cell responses.

Direct CTL functional regulation is exemplified by the lncRNA NKILA-mediated pathway which promotes tumor immune evasion by directly sensitizing CTLs to activation-induced cell death (AICD). NKILA is transcriptionally induced in tumor-infiltrating CTLs upon T-cell receptor stimulation. Research demonstrates that calcium influx activates calmodulin, which removes histone deacetylases (HDAC) from the NKILA promoter to enhance STAT1-mediated transcription. NKILA binds the NF-κB/inhibitor of kappa B (IκB) complex by directly interacting with p65, inhibiting IκBα phosphorylation and preventing its degradation and p65 nuclear translocation. This blockade suppresses Bcl-2-like 1 gene expression, thereby sensitizing CTLs to AICD. Accordingly, NKILA overexpression correlates with CTL apoptosis and poor survival in lung cancer, while its knockdown enhances tumor clearance in patient-derived xenografts (37). Thus, NKILA orchestrates direct CTL dysfunction by epigenetically tuning T-cell sensitivity to apoptotic signals within the immunosuppressive TME.

Beyond intrinsic regulation, immune checkpoint subversion represents a critical tumor escape paradigm. The lncRNA X inactive specific transcript (XIST), previously implicated in breast cancer immune evasion, orchestrates immunosuppression in lung cancer via the XIST/miR-34a-5p/PD-L1 axis. XIST-mediated downregulation of miR-34a-5p elevates PD-L1 expression, directly inhibiting CD8^+^ T cell cytotoxicity (38).

Concurrently, lncRNA-mediated crosstalk with innate immunity reshapes CTL responses through multifaceted mechanisms. LINC00973 mediates complement-dependent immune evasion. Upregulated by epidermal growth factor receptor (EGFR)/the wingless-related integration site (Wnt) signaling, LINC00973 functions as a ceRNA to sequester miR-216b and miR-150, leading to elevated expression of complement inhibitors CD55 and CD59 on tumor cells. These surface proteins block complement-mediated tumor lysis and suppress IL-2/IFN-γ secretion required for CTL activation. Remarkably co-targeting LINC00973 and PD-1 triggers synergistic tumor regression in lung cancer models by reactivating complement-dependent cytotoxicity and CTL function (39). Besides, other lncRNAs like prostate cancer-associated ncRNA transcripts 1 (PCAT1) achieve immunosuppression through distinct pathways, notably by interfering with innate immune sensing. PCAT1 orchestrates immunosuppression via metabolic-immune crosstalk. Mechanistically, in NSCLC, PCAT1 activates the transcription factor SRY-Box transcription factor 2 (SOX2), which directly represses cyclic GMP-AMP synthase (cGAS) promoter activity. Consequently, suppressed cGAS/STING signaling reduces type I interferon production, impairing dendritic cell maturation and CD8^+^ T cell priming. From a therapeutic perspective, PCAT1 inhibition restores radiotherapy-induced immune activation and synergizes with anti-PD-L1 in preclinical models (40). However, the unresolved mechanisms of optimally combining PCAT1 targeting with radiotherapy to modulate immunity represent a prime opportunity for exploring novel combinatorial immunotherapies (40).

In this regard, these findings demonstrate that lncRNA dysregulation promotes tumor immune evasion through both direct CTL functional regulation and multifaceted crosstalk with innate immunity, positioning them as promising targets for immunotherapy, particularly in combination with checkpoint blockade.

#### 3.1.2 Innate immune cells

**Figure 5:**
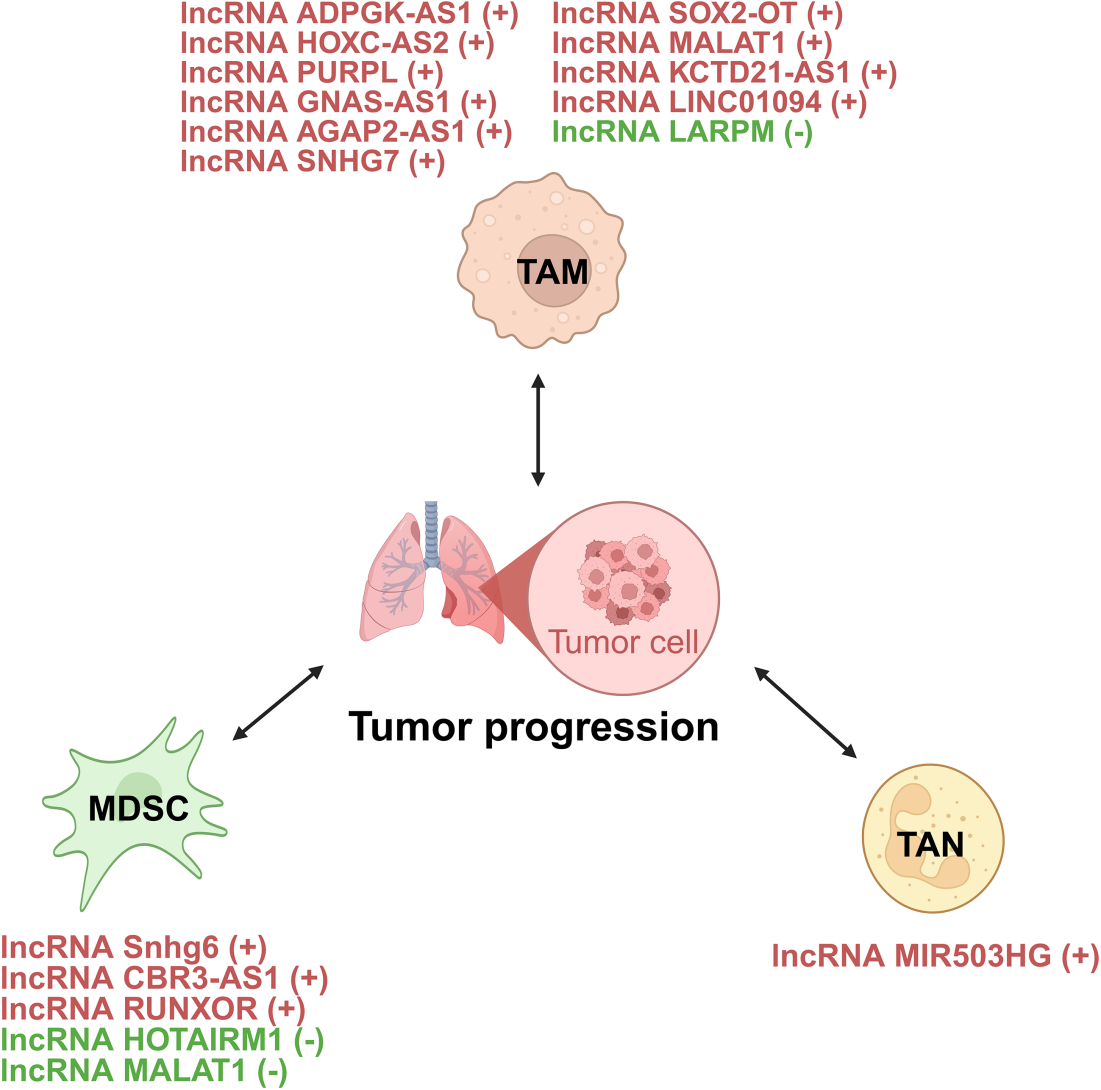
lncRNAs act as modulators between innate immune cells and tumor cells of lung cancer. +: promoting the tumor progression; -: inhibiting the tumor progression. TAM, tumor-associated macrophage; MDSC, myeloid-derived suppressor cell; TAN, tumor-associated neutrophil.

Innate immune cells include TAMs, MDSCs, and TANs. They interact with lncRNAs in the lung TME and affect the formation of immunosuppressive TME (**Figure 5**).

##### 3.1.2.1 TAMs

**Figure 6:**
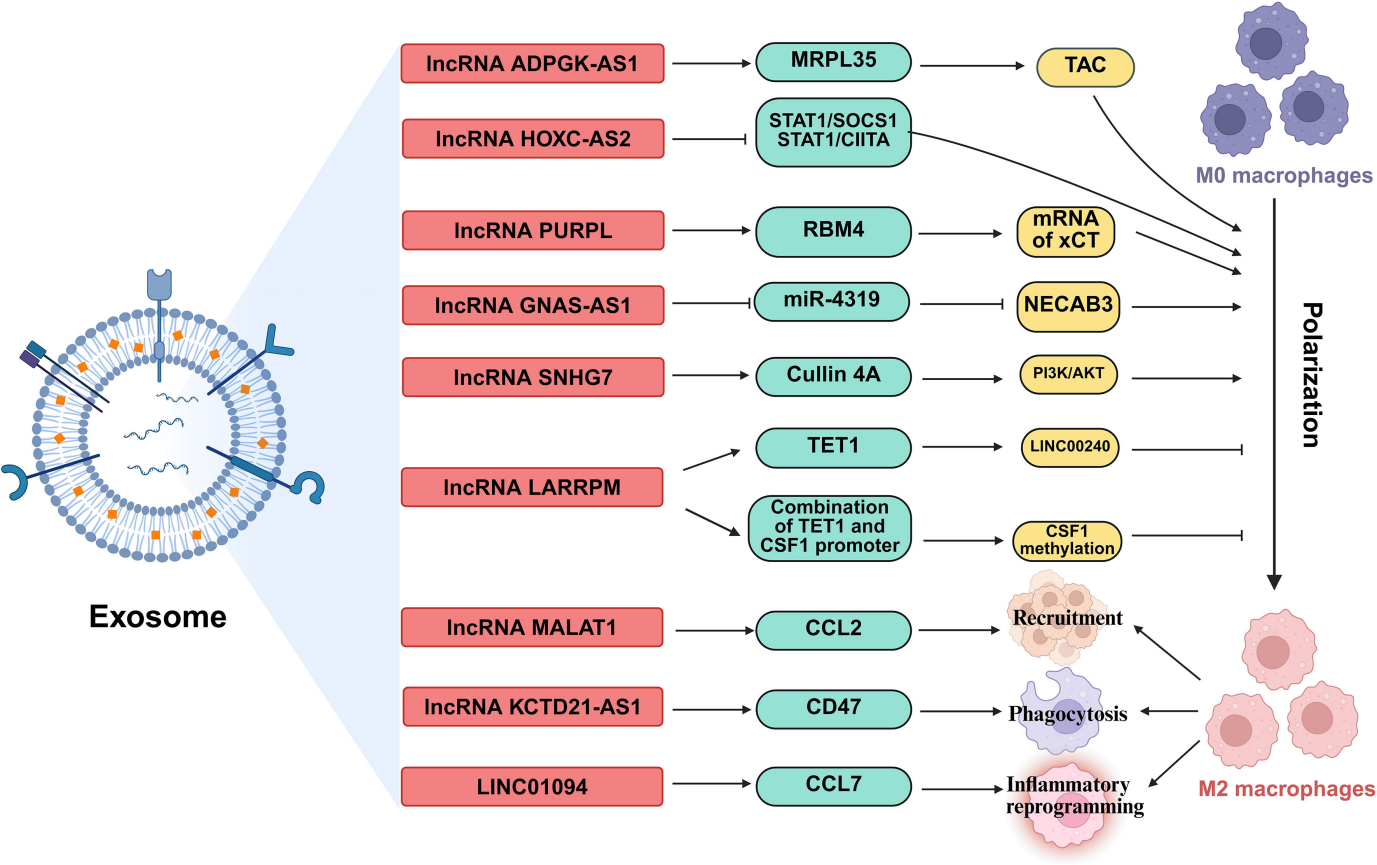
The regulatory roles of lncRNAs in TAMs. lncRNAs, shuttled via exosomes, are involved in regulation the polarization of TAMs, orchestrating TAM recruitment, phagocytic capacity, and inflammatory reprogramming. MRPL35, Mitochondrial ribosomal protein L35; STAT1, Signal transducer and activator of transcription 1; SOCS1, Suppressor of cytokine signaling 1; CIITA, class II transactivator; RBM4, RNA-binding motif protein 4; TET1, Ten-eleven translocation 1; CSF1, Colony stimulating factor 1; CCL, C-C motif chemokine ligand; NECAB3, N-terminal EF-hand calcium binding protein 3.

TAMs, key components of the TME, can both suppress and promote tumor progression (88). They show phenotypic plasticity, polarizing into proinflammatory (M1-like) or anti-inflammatory (M2-like) phenotypes based on stimuli. While TAMs may initially inhibit tumors, they often acquire an M2-like phenotype as tumors grow, supporting tumor progression (89). They are linked to processes like angiogenesis, immunosuppression, metastasis, and therapy resistance, but can also have antitumor effects via phagocytosis and immune promotion (89). lncRNAs critically regulate TAMs in lung cancer through directly controlling macrophage polarization states, mediating intercellular crosstalk via exosomal transfer, and orchestrating TAM recruitment, phagocytic capacity, and inflammatory reprogramming (90) (**Figure 6**).

lncRNAs orchestrate macrophage polarization in lung cancer, tipping the balance between anti-tumor M1 and pro-tumor M2 states (91). For instance, the lncRNA ADPGK antisense RNA 1 (ADPGK-AS1) is substantially upregulated in M2-like macrophages and TAMs. Mechanistically, ADPGK-AS1 localizes to mitochondria, binds mitochondrial ribosomal protein MRPL35, enhances the tricarboxylic acid cycle (TAC), and promotes mitochondrial fission. This metabolic shift drives a phenotypic switch toward tumor-promoting M2-like states, facilitating lung cancer growth. Significantly, macrophage-specific ADPGK-AS1 knockdown reverts cells to tumor-suppressive M1-like states, inhibiting tumor progression across preclinical models (41). In contrast, LINC00240 antisense RNA regulating promoter methylation (LARRPM) is downregulated in advanced lung adenocarcinoma (LUAD) and correlates with poor prognosis. At the molecular level, LARRPM recruits the DNA demethylase ten-eleven translocation methylcytosine dioxygenase 1 (TET1) to the promoter of its antisense gene LINC00240, reducing DNA methylation and activating LINC00240 transcription. Simultaneously, LARRPM decreases TET1 binding to the colony-stimulating factor 1 (CSF1) promoter, increasing CSF1 methylation and repressing this key M2-polarization factor. Collectively, these epigenetic actions restrict M2 TAM polarization and LUAD progression (42). The potential influence of LARRPM on other TET1 targets remains unclear, underscoring the need to fully elucidate its context-specific regulatory mechanisms (42). Recently, additional lncRNAs skew macrophages toward M2. For example, lncRNA HOXC cluster antisense RNA 2 (HOXC-AS2) silences STAT1 signaling (43), lncRNA p53 upregulated regulator of p53 levels (PURPL) upregulates cystine glutamate reverse transporter (xCT)-mediated programs (44), and lncRNA GNAS antisense RNA 1 (GNAS-AS1) relieves miR-4319 repression of N-terminal EF-hand calcium binding protein 3 (NECAB3) (45). Together, these findings outline a druggable lncRNA network that governs TAM fate and tumor progression.

Exosomes serve as critical vehicles for lncRNA-mediated crosstalk between tumor cells and TAMs (92). As an example, M2 macrophage-derived exosomal lncRNA AGAP2 antisense RNA 1 (AGAP2-AS1) transferred to lung cancer cells. Upon delivery, AGAP2-AS1 functions as a ceRNA, sequestering miR-296 to derepress its target notch homolog protein 2 (NOTCH2). This axis ultimately enhances tumor cell radioresistance and dampens radiotherapy efficacy. Therapeutically, silencing exosomal AGAP2-AS1 restores radiation sensitivity, highlighting its role as an intercellular effector of treatment resistance (46). However, the functional relevance of this proposed ceRNA model for AGAP2-AS1 remains to be fully elucidated (46). Likewise, tumor-derived exosomal lncRNA small nucleolar RNA host gene 7 (SNHG7) promotes docetaxel resistance in LUAD. Mechanistically, exosomal SNHG7 is internalized by recipient cells where it recruits Human Antigen R (HuR) to stabilize autophagy genes autophagy related 5 (ATG5) and autophagy related 12 (ATG12), inducing protective autophagy, and activates the PI3K/AKT pathway by recruiting Cullin 4A to degrade the tumor suppressor phosphatase and tensin homolog (PTEN), thereby driving M2 macrophage polarization. As a result, SNHG7 fosters a dual chemoresistance mechanism in tumor cells and their surrounding immune landscape (47). NSCLC cell-derived exosomal lncRNA SOX2 overlapping transcript (SOX2-OT) also induces M2 polarization, contributing to epidermal growth factor receptor-tyrosine kinase inhibitor (EGFR-TKI) resistance (48). These findings revealed that tumor and TAMs use exosomal lncRNAs as “molecular messages” to jointly promote therapy resistance and immune suppression.

Beyond polarization and exosomes, lncRNAs directly orchestrate TAM recruitment, phagocytic capacity, and inflammatory reprogramming. A key regulator is the metastasis-associated lncRNA metastasis-associated LUAD transcript 1 (MALAT1), which is overexpressed in LUAD. Mechanistically, MALAT1 induces global chromatin remodeling, increasing accessibility at the CCL2 promoter. This action elevates CCL2 secretion, recruiting protumorigenic macrophages to the TME. Critically, MALAT1-driven metastasis is abrogated by CCL2 blockade or macrophage depletion *in vivo*, establishing its non-cell-autonomous role in shaping the immunosuppressive niche (49). However, whether MALAT1 overexpression during later stages of tumor development exerts the same potent pro-metastatic effect as during initiation remains to be determined (49). Furthermore, the N6-methyladenosine (m^6^A)-modified lncRNA KCTD21 antisense RNA 1 (KCTD21-AS1) impairs macrophage phagocytosis by upregulating the “don’t eat me” signal CD47 and promoting autophagy via TIP41-like protein (50). LINC01094 further exemplifies chemokine-mediated crosstalk, promoting SPI1-dependent chemokine ligand 7 (CCL7) transcription to recruit M2 macrophages and facilitate metastasis (51). Together, these lncRNAs act as central regulators that control where TAMs gather, how they function, and their role in supporting tumor growth.

Taken together, these findings demonstrate that lncRNAs critically orchestrate the pro-tumorigenic functions of TAMs in lung cancer through multifaceted mechanisms. These lncRNA-driven alterations profoundly contribute to tumor progression, immunosuppression, and treatment failure, positioning them as compelling therapeutic targets, potentially in combination with existing modalities.

##### 3.1.2.2 MDSCs

MDSCs are heterogeneous cells from immature myeloid progenitors. They promote lung cancer progression by creating an immunosuppressive TME. These cells inhibit antitumor immune responses and promote tumor progression by facilitating tumor angiogenesis, tumor cell invasion, and premalignant niche formation. High levels of MDSCs are associated with poor clinical outcomes and increased resistance to chemotherapy and immunotherapy in lung cancer patients (24). Recent studies have highlighted the regulatory role of lncRNAs in the function and differentiation of MDSCs in lung cancer. These lncRNAs can either enhance or suppress the immunosuppressive activity of MDSCs, thereby influencing tumor progression and therapeutic outcomes (93).

Certain lncRNAs, such as HOXA transcript antisense RNA, myeloid-specific 1 (HOTAIRM1) and MALAT1, have been shown to suppress the immunosuppressive activity of MDSCs. Specifically, HOTAIRM1, a lncRNA crucial for myeloid cell differentiation, is significantly downregulated in MDSCs isolated from lung cancer patients. At the molecular level, HOTAIRM1 is associated with elevated expression of the transcription factor homeobox A1 (HOXA1). Intriguingly, elevated HOXA1 gene levels curtail the immunosuppressive function of MDSCs. However, the precise mechanism underlying this regulation in MDSCs remains to be fully elucidated (52). In a comparable manner, MALAT1, a widely studied lncRNA, exhibits downregulation in peripheral blood mononuclear cells of lung cancer patients. Consistently, low MALAT1 expression correlates with a higher proportion of circulating MDSCs, and its experimental knockdown increases MDSC frequency, indicating a negative regulatory role for MALAT1 in MDSC accumulation (94). However, its regulatory mechanism needs to be further studied in future researches. All together, these lncRNAs act as endogenous brakes that constrain MDSC-mediated immunosuppression.

On the flip side, some lncRNAs contribute to MDSC-mediated immunosuppression. lncRNA SNHG6 is highly expressed in tumor-derived MDSCs within Lewis lung carcinoma models (53). Mechanistically, SNHG6 facilitates the differentiation of monocytic MDSCs (M-MDSCs) by promoting the ubiquitination and subsequent degradation of EZH2, a key epigenetic regulator. While SNHG6 primarily influences M-MDSC differentiation without directly altering their established immunosuppressive function, its action enhances the plenty of immunosuppressive MDSCs within the TME, contributing to tumor progression (53). Another prominent example involves RNA binding motif protein 15 (RBM15) and the lncRNA CBR3 antisense RNA 1 (CBR3-AS1). Research demonstrates that RBM15, potentially through mediating m6A modification, stabilizes the lncRNA CBR3-AS1 (54). In terms of its functional role, lncRNA CBR3-AS1 acts as a ceRNA, sponging miR-409-3p. This sequestration relieves miR-409-3p-mediated repression of its target, C-X-C motif chemokine ligand 1 (CXCL1). Consequently, elevated CXCL1 expression recruits MDSCs to the tumor site. Critically, this RBM15/CBR3-AS1/miR-409-3p/CXCL1 axis not only recruits MDSCs but also inhibits T cell activity, thereby promoting radioresistance in NSCLC (54). While this work highlights a direct link between lncRNA-mediated MDSC regulation and therapeutic response, its clinical implications warrant further validation, particularly given the potential confounding effects of concurrent chemotherapy in the study cohort and the current absence of targeted inhibitors for this pathway (54). Similarly, lncRNA RUNX1 overlapping RNA (RUNXOR) is elevated in both the peripheral blood and MDSCs of lung cancer patients. By repressing its overlapping gene RUNX1, RUNXOR upregulates Arg-1, the signature immunosuppressive enzyme of MDSCs, and thereby fosters tumor immune escape (55). However, that the precise molecular mechanism by which RUNXOR regulates the RUNX1-Arg1 axis in MDSCs remains to be fully elucidated (55). Therefore, such lncRNAs function as molecular accelerators that drive MDSC-dependent immune evasion.

Collectively, these findings underscore lncRNAs as pivotal regulators of MDSC abundance and immunosuppressive function in the lung TME. However, it is noteworthy that for many of these lncRNAs, such as HOTAIRM1 and RUNXOR, the precise molecular mechanisms, including their exact binding partners or downstream effectors, remain incompletely characterized. A deeper mechanistic understanding will be crucial for evaluating their potential as therapeutic targets.

##### 3.1.2.3 TANs

Neutrophils contribute to cancer progression by inducing oxidative DNA damage and promoting tumor cell proliferation in TME (95). A key mechanism underlying their pro-tumorigenic activity is the formation of NETs. These structures, composed of chromatin DNA and granule proteins, facilitate lung cancer metastasis by accumulating at distant sites, where their DNA component acts as a chemotactic factor to promote cancer cell adhesion and seeding (96). The role of lncRNAs in regulating NET in lung cancer is an emerging area of research (95). However, research on lncRNAs specifically regulating NETs in lung cancer remains scarce. The lncRNA MIR503 host gene (MIR503HG) serves as a prominent example, with studies demonstrating how TAN-derived NETs subvert its function to promote metastasis (56, 97, 98).

First establishing MIR503HG’s tumor-suppressive role, Lin et al. documented consistent downregulation of this lncRNA in NSCLC tissues and cell lines. Functional assays demonstrated that MIR503HG overexpression inhibited proliferation and induced apoptosis, primarily through suppressing Wnt1 expression. Crucially, xenograft models confirmed reduced tumor growth, positioning MIR503HG as a metastasis suppressor (97). Based on this, Wang et al. discovered that NETs in the lung TME downregulate the MIR503HG. This downregulation was identified by lncRNA microarray analysis and confirmed by qRT-PCR. They found that NET exposure promotes metastasis by inducing EMT in NSCLC cells. Mechanistically, NET-mediated suppression of MIR503HG leads to hyperactivation of the NF-κB signaling pathway, which in turn triggers the NLRP3 inflammasome axis. Ultimately, this signaling cascade drives lung cancer dissemination. Crucially, rescue experiments demonstrated that restoring MIR503HG expression effectively blocked NET-facilitated metastasis in mouse models (56). The precise mechanism by which NETs downregulate MIR503HG remains unclear. Further study by Ye et al. decoded the full TAN/NET-MIR503HG-NLRP3 axis driving NSCLC metastasis. NETs silence the lncRNA MIR503HG via promoter methylation. This prevents MIR503HG from facilitating ring finger protein 43 (RNF43)-mediated ubiquitination and degradation of the transcription factor CCAAT/enhancer binding protein beta (C/EBPβ). Accumulated C/EBPβ then activates the NLRP3 promoter, elevating inflammasome activity and metastasis. DNA methyltransferase inhibitors reversed this effect, restoring MIR503HG and suppressing metastasis (98). However, these mechanistic insights are primarily derived from *in vitro* models of NETs. These findings require validation in more physiologically relevant *in vivo* systems to fully capture the complexities of the intact TME (98).

Collectively, TANs release NETs that suppress MIR503HG expression. This silencing activates key pro-metastatic cascades like NF-κB/NLRP3 inflammasome hyperactivation, ultimately driving lung cancer dissemination (56, 97, 98). Furthermore, building upon this established axis, investigating whether other lncRNAs are similarly regulated by NETs, or whether TANs themselves export regulatory lncRNAs via extracellular vesicles to regulate other cells in the niche, represents a promising research direction.

#### 3.1.3 Other tumor associated cells

##### 3.1.3.1 CSCs

**Figure 7:**
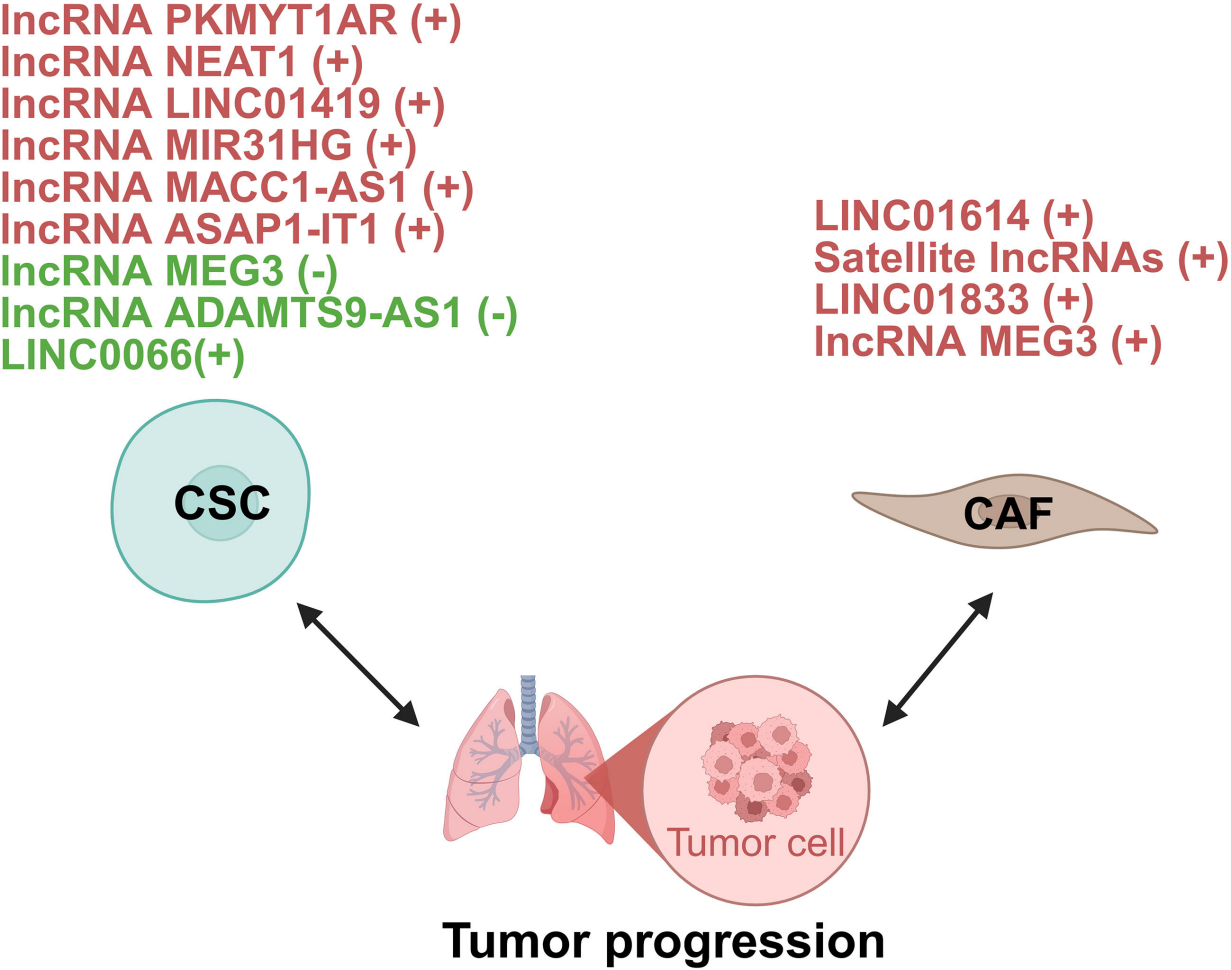
lncRNAs act as modulators between CSC/CAF and lung tumor cells. +: promoting the tumor progression; -: inhibiting the tumor progression. CSC, cancer stem cell; CAF, cancer-associated fibroblasts.

CSCs are vital in tumor biology, driving initiation, progression, metastasis, and therapy resistance. They self-renew and differentiate, initiating tumors and maintaining heterogeneity. CSCs proliferate to fuel growth, invade via EMT, and resist therapy through dormancy and drug efflux. They evade immunity by creating a suppressive microenvironment and reactivating post-treatment to cause recurrence (99). lncRNAs regulate lung cancer CSCs through multiple mechanisms to modulate their maintenance and stemness (**Figure 7**).

In NSCLC, lncRNAs function as master regulators of CSC plasticity by modulating developmental signaling pathways. Central to Wnt/β-catenin hyperactivation is the human-specific PKMYT1 associated lncRNA (PKMYT1AR) which is induced by Yin Yang 1 factor (YY1). It sequesters tumor-suppressive miR-485-5p to stabilize PKMYT1, thereby inhibiting β-catenin degradation and driving CSC maintenance. Crucially, antisense oligonucleotide (ASO)-mediated PKMYT1AR silencing suppresses xenograft tumorigenesis, underscoring its therapeutic promise (57). However, the molecular function of PKMYT1 itself appears to be highly context-dependent, as it stabilizes β-catenin through distinct mechanisms in different cancers (57). Simultaneously, lncRNA nuclear enriched abundant transcript 1 (NEAT1) amplifies this Wnt-driven axis by inhibiting copper transporter 1 and potentiating EMT cascades (58). Beyond Wnt signaling, LINC01419 recruits EZH2 to epigenetically silence fructose-1,6-bisphosphatase 1 (FBP1), activating Hippo signaling in CD44^+^ LUAD cells and conferring chemoresistance (59).

Epigenetic reprogrammers mediate CSC plasticity through chromatin remodeling. MIR31HG is elevated in NSCLC patient sera and correlates with poor prognosis. Mechanistically, MIR31 host gene (MIR31HG) recruits the WD repeat-containing protein 5 (WDR5)/mixed lineage leukemia 3 (MLL3)/P300 complex to deposit activating histone marks including histone H3 lysine 4 mono-methylation (H3K4me1) and histone H3 acetylated lysine 27 (H3K27ac) at the GLI2 promoter, augmenting transcription of this Hedgehog pathway effector. Functionally, this confers CSC-like properties including chemoresistance and metastasis. Intriguingly, *in vivo* ASOs targeting of MIR31HG attenuates metastasis, positioning it as a diagnostic biomarker and therapeutic target (60). Further research is needed to define the genomic targeting scope of MIR31HG and to overcome the translational challenge posed by its lack of a murine homolog.

Another group of lncRNAs enhance CSC stemness through RNA interactions. For instance, lncRNA MACC1 antisense RNA 1 (MACC1-AS1) contributes to CSC stemness by interacting with up-frameshift 1 (UPF1) to destabilize large tumor suppressor kinase (LATS) 1/2 mRNA, further regulating the Hippo signaling pathway (61). In addition, lncRNA ASAP1 intronic transcript 1 (ASAP1-IT1) sponges miR-509-3p to derepress yes-associated protein 1 (YAP1), thus activating the Hippo pathway effector and enhancing cancer stemness in NSCLC (62). Moreover, LINC00662 promotes lung cancer cell invasion and CSC-like phenotypes by interacting with Lin28, highlighting its potential as a diagnostic and therapeutic target (63). Collectively, these studies demonstrate that lncRNA-mediated RNA interactions represent a versatile mechanism sustaining cancer stemness properties.

On the other hand, some lncRNAs act as suppressors of CSC characteristics. MEG3, downregulated in lung cancer stem cells (LCSCs), suppresses migration and invasion by sponging miR-650, which targets solute carrier family 34 member 2 (64). lncRNA ADAMTS9 antisense RNA 1 (ADAMTS9-AS1) is downregulated in LUAD and linked to better survival. It suppresses LUAD cell stemness by upregulating miR-5009-3p, which targets nephronectin. Overexpressing ADAMTS9-AS1 curbs LUAD progression *in vitro* (65).

lncRNAs orchestrate CSC plasticity through multi-tiered regulation, including signaling pathway activation, epigenetic reprogramming, and RNA interactions. This integrated network sustains stemness and immunosuppression, positioning lncRNAs as master regulators of CSC resilience.

##### 3.1.3.2 CAFs

CAFs are key players in the TME, interacting with cancer and stromal cells to promote tumor progression, metastasis, and chemoresistance. Their significant heterogeneity and plasticity, stemming from diverse origins, necessitates understanding their diversity for effective therapies (100). The functional output of CAFs is critically regulated by lncRNAs, which act as master switches controlling their activation and matrix-remodeling capabilities. These lncRNAs operate through several core mechanisms, such as driving glycolytic reprogramming to trigger fibronectin secretion (101), stabilizing key cytokines to sustain an autocrine loop that maintains CAFs in an activated state (102), and directly hyperactivating signaling pathways like AKT (103) to orchestrate the transcriptional program for collagen deposition and stromal stiffening. In lung cancer, lncRNAs act as critical regulators of CAF functions, modulating tumor metabolism and transcriptional programs (**Figure 7**).

Emerging evidence highlights lncRNAs within CAFs as key orchestrators of metabolic reprogramming in cancer cells. A prime example identified in LUAD is the CAF-specific lncRNA LINC01614, which drives glutamine metabolic reprogramming. Mechanistically, LINC01614 is packaged into CAF exosomes and transferred to tumor cells, where it binds annexin A2 (ANXA2) and p65 to activate NF-κB signaling. This reprograms glutamine metabolism by upregulating transporters solute carrier family 38 member 2 (SLC38A2) and solute carrier family 7 member 5 (SLC7A5), boosting glutamine uptake to fuel tumor growth. Critically, tumor-derived cytokines further induce LINC01614 in CAFs, creating a feedforward loop. Blocking exosomal transfer suppresses glutamine addiction and tumor progression (66). This study highlights the therapeutic potential of targeting a CAF-specific lncRNA to inhibit glutamine utilization and cancer progression in LUAD.

Beyond specific metabolites, CAFs can induce broad transcriptional changes via satellite lncRNAs. Pericentromeric satellite lncRNAs are robustly induced in CAFs within the LUAD TME. These lncRNAs are transcriptionally activated in response to diverse stromal stimuli, including TGF-β, IL-1α, increased matrix stiffness, direct contact with tumor cells, and exposure to chemotherapeutic drugs. Following their induction, satellite lncRNAs are localized not only within the CAF nucleus and cytoplasm but are also packaged into extracellular vesicles (EVs) for secretion. Functionally, knockdown experiments revealed that these satellite lncRNAs are essential for maintaining an inflammatory CAF phenotype. Depleting satellite transcripts attenuates cellular senescence in CAFs and disrupts their ability to adopt a pro-tumorigenic, immunosuppressive state. This impairment consequently inhibits the pro-tumorigenic functions of CAFs (67). These findings position pericentromeric satellite lncRNAs as novel regulators of CAF activation and mediators of intercellular communication within the TME, presenting potential biomarkers or targets.

Similar mechanisms of CAF-derived exosomal lncRNAs driving tumor progression exist. For instance, exosomal LINC01833 from CAFs promotes NSCLC cell proliferation, migration, and invasion by acting as a ceRNA for miR-335-5p, leading to increased VAMP associated protein A (VAPA) expression (68). Likewise, CAF-derived exosomal lncRNA maternally expressed 3 (MEG3) binds miR-15a-5p in small cell lung cancer (SCLC) cells, derepressing cyclin E1 (CCNE1) to drive cisplatin chemoresistance and tumor progression (69).

In summary, CAF-derived exosomal lncRNAs mediate tumor-stroma communication to control cancer metabolism and transcriptional programs, critically driving lung cancer progression.

### 3.2 lncRNAs in immune checkpoint regulation

Immune checkpoints regulate immune activity and can be exploited by tumors to evade attack, creating an immunosuppressive microenvironment. Key checkpoints like PD-1/PD-L1 and CTLA-4 are critical targets. Tumor-intrinsic factors and oncogenic pathways further enable immune evasion (29). Critically, lncRNAs are emerging as master regulators of these checkpoints, particularly PD-L1, in lung cancer. Their control spans two key levels, including PD-L1 transcription and mRNA stability, PD-L1 protein stability and isoform generation.

Transcriptional control by lncRNAs constitutes a pivotal mechanism exploited by environmental carcinogens to drive oncogenesis. Hexavalent chromium [Cr (VI)], a potent lung carcinogen, triggers overexpression of the lncRNA ABHD11 antisense RNA 1 (ABHD11-AS1) in lung epithelial cells. Functionally, ABHD11-AS1 recruits the deubiquitinase ubiquitin specific peptidase 15 (USP15) to degrade tumor necrosis factor receptor-associated factor 3 (TRAF3), thereby activating the non-canonical NF-κB pathway. Consequently, this cascade amplifies IL-6/STAT3 signaling and robustly upregulates PD-L1 transcription. Critically, ABHD11-AS1-driven PD-L1 elevation suppresses T cell infiltration and accelerates lung carcinogenesis in murine models, positioning this lncRNA as a molecular bridge between environmental exposure and immune evasion (70). Once PD-L1 mRNA is made, its half-life can be prolonged by lncRNA SWI/SNF complex antagonist associated with prostate cancer 1 (SChLAP1), which recruits AU-rich element RNA-binding factor 1 (AUF1) to the 3′-UTR and shields the transcript from degradation, thereby elevating steady-state protein levels and fueling tumor growth (71). These studies indicate that CAF-derived lncRNAs emerge as direct transcriptional regulators of pro-tumorigenic programs.

Beyond transcriptional regulation, lncRNAs critically govern PD-L1 protein stability and generate functionally diversified isoforms through distinct pathways. In NSCLC, the m^6^A-modified lncRNA LINC02418 directly controls PD-L1 protein stability by scaffolding tripartite motif containing 21 (TRIM21) to induce PD-L1 ubiquitination and proteasomal degradation. Silencing of this tumor suppressor elevates PD-L1 abundance, while its restoration enhances anti-PD-L1 therapy efficacy (72). The PD-L1 gene also expresses a long non-coding splice variant (PD-L1-lnc). Upon IFN-γ induction, this lncRNA promotes LUAD progression by directly enhancing c-Myc transcriptional activity (73). Targeting PD-L1-lnc holds exceptional therapeutic promise by simultaneously disrupting dual oncogenic-immune pathways.

These studies collectively delineate lncRNAs as central orchestrators of immune evasion pathways in lung cancer, operating through diverse mechanisms that converge on suppressing anti-tumor immunity.

### 3.3 lncRNAs in cytokines and immune modulators

lncRNAs serve as pivotal regulators of cytokine networks within TME, fine-tuning immune responses and stromal activation (104, 105). Among the cytokines and immune modulators influenced by lncRNAs, the TGF-β is the most extensively studied in lung cancer. We will therefore focus on TGF-β, while also noting other cytokine modulations by lncRNAs.

#### 3.3.1 lncRNAs in regulation of TGF-β

TGF-β plays a complex dual role in lung carcinogenesis (106). In early-stage NSCLC, it acts as a tumor suppressor by inhibiting epithelial cell proliferation. However, in advanced disease, TGF-β becomes a potent driver of tumor progression through multiple mechanisms (107). It is the primary inducer of EMT in NSCLC cells, enhancing migratory and invasive capacities while conferring anti-apoptotic properties and chemoresistance (108). This process is markedly amplified by oncogenic kirsten ratsarcoma viral oncogene homolog (KRAS) signaling (109). Beyond direct cancer cell effects, TGF-β critically shapes TME by activating CAFs, thereby promoting extracellular matrix remodeling and tissue stiffening (110). It also facilitates angiogenesis and orchestrates immune evasion through Treg induction, polarization of tumor-associated immune cells, and exclusion of cytotoxic T lymphocytes. In SCLC, TGF-β signaling is frequently disrupted, particularly in classic subtypes lacking TGFβR-II expression. This inactivation derepresses the neuroendocrine master regulator achaete-scute family bHLH transcription factor 1 (ASCL1), contributing to SCLC pathogenesis by maintaining the neuroendocrine phenotype and potentially enhancing cell survival (111).

**Figure 8:**
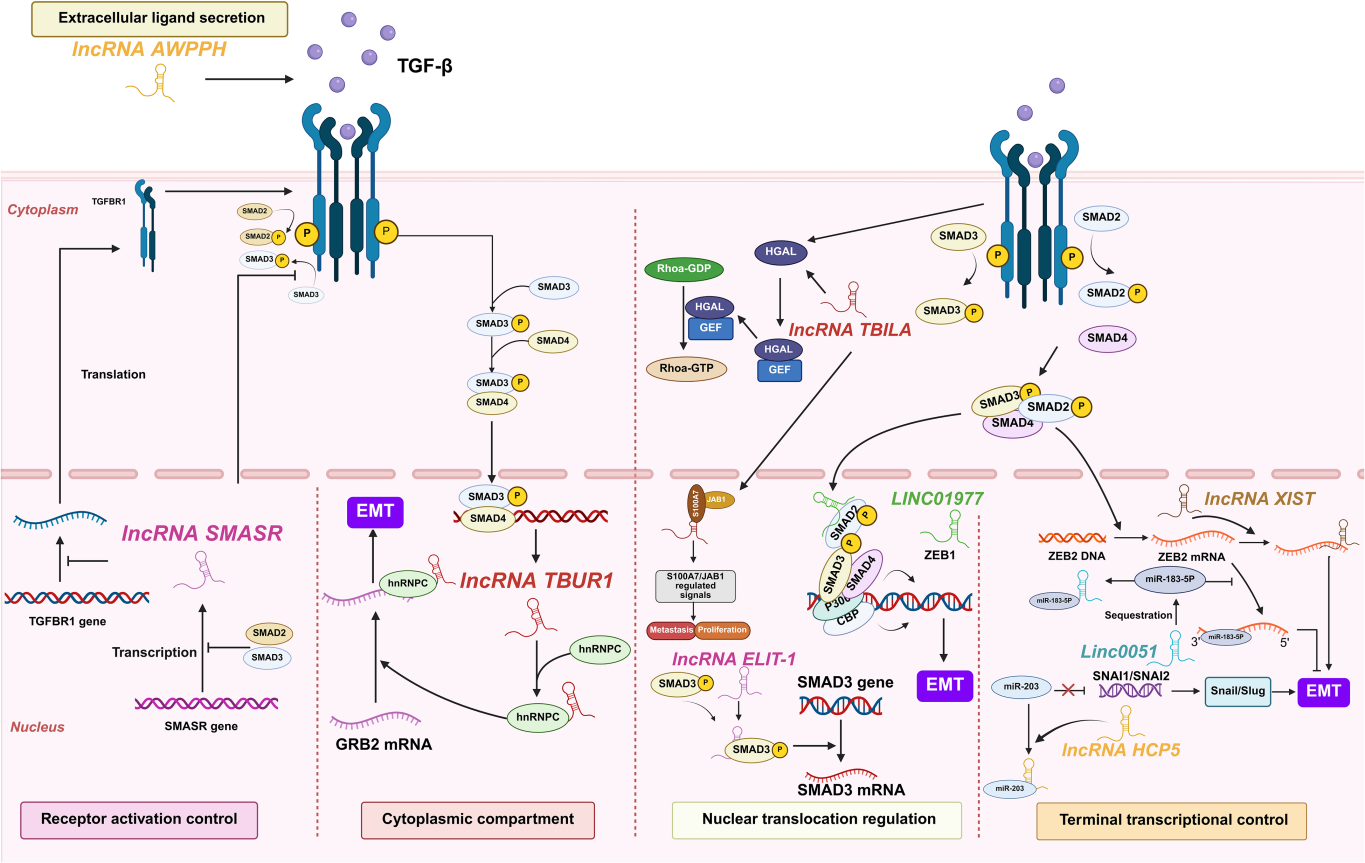
lncRNAs act as modulators of TGF-β in lung cancer. Lung cancer-associated lncRNAs exert sequential control over TGF-β signaling, coordinating five regulatory tiers from extracellular ligand engagement to nuclear transcriptional activation, collectively promoting tumor progression and immune evasion.

Lung cancer-associated lncRNAs execute sequential control of TGF-β signal transduction, coordinating molecular events from extracellular ligand engagement to nuclear transcriptional activation (**Figure 8**).

##### 3.3.1.1 Extracellular ligand engagement

At the extracellular ligand secretion level, lncRNA associated with poor prognosis of hepatocellular carcinoma (AWPPH) directly elevates TGF-β1 production in resected NSCLC tumors. Clinical evidence reveals that blood AWPPH levels are positively correlated with TGF-β1 mRNA in distant recurrence patients, and AWPPH overexpression experimentally upregulates TGF-β1 protein expression. This establishes an autocrine feedforward loop that drives postoperative metastasis through sustained TGF-β1 secretion (74). However, the precise mechanism by which AWPPH upregulates TGF-β1 remains unknown.

##### 3.3.1.2 Receptor activation control

For receptor activation control, SMAD3 associated lncRNA (SMASR) suppresses TGF-β receptor activation through binding unphosphorylated SMAD2/3 to block their phosphorylation while concurrently inhibiting TGFBR1 transcription. This negative feedback circuit is initiated by TGF-β itself, which downregulates SMASR expression via SMAD2/3, thereby constraining signal initiation at the plasma membrane (75). However, these proposed mechanisms require further validation. Key unresolved questions include identifying the specific SMAD domains critical for SMASR binding and exploring its potential non-transcriptional functions in the cytoplasm (75).

##### 3.3.1.3 Cytoplasmic signal transduction

Within the cytoplasmic compartment, lncRNAs critically amplify TGF-β signaling through stabilizing core pathway components. TGF-β/SMAD3/4-activated TGF-β-upregulated lncRNA 1 amplifies cytoplasmic signal propagation by binding heterogeneous nuclear ribonucleoprotein C (hnRNPC) to stabilize growth factor receptor bound protein 2 (GRB2) mRNA in an m^6^A-dependent manner. This stabilization potentiates ERK phosphorylation and downstream MAPK signaling, thereby driving EMT initiation, enhancing LUAD cell migration/invasion *in vitro*, and promoting metastasis *in vivo*. Critically, TBUR1 overexpression correlates with poor clinical prognosis in LUAD, positioning it as both a metastasis driver and actionable therapeutic target (76).

##### 3.3.1.4 Nuclear translocation regulation

Nuclear translocation regulation involves LINC01977-mediated hijacking of super-enhancer elements, which facilitates SMAD3 nuclear import and recruits CBP/P300 to the zinc finger e-box binding homeobox 1 (ZEB1) promoter (77). TGF-beta induced lncRNA (TBILA), induced by TGF-β, binds nuclear SMAD complexes to enhance RhoA activation via cis-regulating human germinal center-associated lymphoma (HGAL) expression. Concurrently, it activates the S100A7-JAB1 pro-survival pathway by interacting with nuclear S100A7, facilitating NSCLC metastasis (78). Nuclear-localized lncRNA enforcing TGF-β signaling 1 (LETS1) potentiates TGF-β-SMAD signaling by stabilizing cell surface TβRI. Specifically, LETS1 binds nuclear factor of activated T cells (NFAT5) and induces expression of orphan nuclear receptor subfamily 4 group A member 1 (NR4A1), which targets SMAD7 for degradation in a destruction complex. This mechanism inhibits SMAD7-mediated polyubiquitination of TβRI, extending receptor half-life and enhancing TGF-β signal transduction. Crucially, TGF-β transcriptionally induces LETS1 expression, creating a nuclear-originated positive feedback loop that promotes EMT and cancer cell extravasation (79).

##### 3.3.1.5 Nuclear transcriptional activation

Terminal transcriptional control executes TGF-β signaling through the following lncRNA mechanisms. Human histocompatibility leukocyte antigen complex p5 (HCP5) achieves this control by sequestering miR-203 via direct binding, lifting SNAI1/SNAI2 repression to elevate Snail/Slug for EMT initiation (80). Concurrently, EMT-associated lncRNA induced by TGFβ1 (ELIT-1) binds to SMAD3 in nucleus upon TGFβ-stimulation, promoting the transcription of SMAD-target genes by facilitating SMAD3 recruitment to their respective promoters as a SMAD3 cofactor, enforcing terminal control (81). LINC00511 executes terminal transcriptional control by sponging miR-183-5p to derepress zinc finger E-box binding homeobox 2 (ZEB2), directly activating EMT transcription programs and matrix metalloproteinases (MMP) expression, thereby irreversibly committing TGF-β-driven metastasis (82). As a master terminal regulator, lncRNA XIST enforces irreversible EMT execution by ceRNA-mediated ZEB2 derepression, driving TGF-β-dependent transcriptional reprogramming and *in vivo* metastatic colonization, thus locking cells in a pro-metastatic state (83).

Collectively, these findings position lncRNAs as master regulators that exert precise control over the entire TGF-β signaling cascade in lung cancer. Their governance spans from initial ligand availability to the final transcriptional output, critically enforcing the pathway’s switch to a pro-tumorigenic state. Consequently, targeting these specific lncRNA-TGF-β axes presents a promising therapeutic strategy to inhibit TGF-β-mediated tumor progression and immunosuppression.

#### 3.3.2 lncRNAs in regulation of other cytokines and immune regulatory factors

Beyond TGF-β, lncRNAs interact with diverse cytokines and immune regulatory factors including IL-6, IL24 and TNF-α. lncRNA enhancing IL-6/STAT3 signaling activation (LEISA) recruits transcription factor STAT3 to the IL-6 promoter, establishing a feedforward loop that augments IL-6 secretion and STAT3 phosphorylation to promote tumor proliferation and chemotherapy resistance (84). Equally critical for immune evasion, LINC00152 recruits EZH2 to deposit repressive H3K27me3 marks on the IL24 promoter, epigenetically silencing this pro-apoptotic cytokine and enabling uncontrolled tumor growth (85). For TNF-α signaling, TNF-α and heterogenous nuclear ribonucleoprotein L related immunoregulatory lncRNA (THRIL) forms a self-regulatory circuit where TNF-α induces fat mass and obesity-associated (FTO)-mediated m6A demethylation of THRIL, triggering YTH n6-methyladenosine RNA binding protein 2 (YTHDF2)-dependent degradation to suppress NSCLC proliferation. Conversely, THRIL stabilizes TNF-α mRNA via HuR complex formation, establishing a bistable loop that amplifies inflammatory responses while enabling tumor-suppressive feedback at high cytokine concentrations (86).

In summary, lncRNAs exert multifaceted control over the cytokine landscape of the TME, targeting key immune mediators such as IL-6, IL-10, and TNF-α. Future studies should aim to decipher the precise molecular mechanisms through which these lncRNAs govern cytokine networks. Key questions include how they achieve spatial and temporal control over cytokine production and whether their functions are specific to certain cell types. Elucidating these intricate regulatory circuits will be crucial for developing novel therapeutic strategies that target lncRNAs to normalize the immunosuppressive TME and potentially overcome resistance to current immunotherapies.

## 4 The clinical significance of lncRNAs in lung cancer

### 4.1 lncRNAs as clinically stratified biomarkers in TME

lncRNAs are emerging as powerful, clinically stratified biomarkers with significant diagnostic and prognostic utility in lung cancer (112). Crucially, their expression profiles within the TME and detection across diverse, accessible biofluids (including serum, sputum, urine, and pleural effusion) offer unprecedented opportunities for non-invasive diagnosis, precise risk stratification, and microenvironmental assessment (113, 114). Advanced detection techniques enable the robust quantification of these circulating and tissue-derived lncRNAs, solidifying their potential to guide personalized lung cancer management beyond conventional methods (115, 116).

lncRNAs demonstrate significant diagnostic potential across diverse body fluids in lung cancer detection. In serum, elevated levels of XIST and HIF1A antisense RNA 1 (HIF1A-AS1) were consistently observed in NSCLC patients. The combined detection of these lncRNAs demonstrated high diagnostic accuracy, outperforming individual markers (117). Similarly, serum LINC00173 levels were higher in 108 NSCLC patients than in healthy donors and benign pulmonary disease cases. Its combination with carcinoembryonic antigen (CEA) and cytokeratin 19 fragment (Cyfra 21-1) further improved diagnostic accuracy (118). SChLAP1 was significantly upregulated in NSCLC cell lines, serum and tissues, which was identified as an excellent indicator for the diagnosis and prognosis of NSCLC (71). Beyond blood samples, sputum analysis revealed that a panel of SNHG1, H19, and HOTAIR provided high sensitivity and specificity, surpassing conventional sputum cytology for lung cancer detection (119). For urine-based testing, exosomal analysis identified six differentially expressed lncRNAs (70 upregulated and 570 downregulated) through microarray screening, with QT-PCR validation confirming their diagnostic utility (120). Regarding effusion samples, pleural fluid analysis identified small cajal body-specific RNA 7 (SCARNA7), MALAT1, and NONHSAT017369 as biomarkers for EGFR mutation status, with consistent upregulation observed in plasma of EGFR-mutant patients (121). Collectively, these findings firmly establish lncRNAs as clinically actionable biomarkers detectable in serum, sputum, urine, and pleural effusion. Clinical translation now necessitates standardized assays and prospective validation to advance these lncRNAs into routine diagnostic and immunotherapeutic guidance.

lncRNAs demonstrate significant prognostic utility through multidimensional signatures in lung cancer management. Computational frameworks identified TANlncSig, a neutrophil-specific lncRNA signature that independently stratifies patients into distinct survival groups (HR >1, P<0.0001), serving as a marker for myeloid cell infiltration and immunotherapy response (122). Similarly, a 12-NETs-related lncRNA signature predicted shorter overall survival in high-risk patients (P<0.0001), with three adverse lncRNAs validated to increase post-NETs stimulation in tumor cells (123). For TME assessment, the TILSig signature (seven immune infiltration-associated lncRNAs) robustly classified patients into immune-cold and immune-hot groups, where immune-hot patients exhibited prolonged survival and enhanced response to checkpoint inhibitors (124). The LINC00669-based signature combined with tumor stage demonstrated significant prognostic value, stratifying patients into risk subgroups with distinct immune microenvironments and therapy responses (125). For therapy monitoring, CTLA4LncSigs (19 CTLA-4-related lncRNAs) dynamically reflected treatment efficacy, with the risk score serving as an independent prognostic factor (126). Collectively, these signatures enable precise risk stratification, immunotherapy response prediction, and real-time treatment efficacy monitoring beyond conventional imaging methods.

Despite their considerable promise, the clinical translation of lncRNA biomarkers faces several key challenges. A primary limitation is the need for completely noninvasive detection methods, which currently suffer from insufficient sensitivity to reliably detect low-abundance lncRNAs in liquid biopsies. This is compounded by the lack of standardized diagnostic panels with established reference values. Addressing these issues is essential to resolve the pressing question of clinical utility, such as whether these biomarkers can accurately distinguish malignant from benign pulmonary nodules (127).

In summary, lncRNAs demonstrate immense potential as clinically actionable tools for non-invasive diagnosis and multidimensional prognostication in lung cancer. The key biomarkers and signatures discussed above are systematically summarized in **Table 2**.

**Table 2:** lncRNAs as diagnostic and prognostic biomarkers in lung cancer.

Clinical significance	lncRNA	Cancer types	Regulation	Sample types	Function	Sensitivity	Specificity	Ref.
Diagnostic biomarker	XIST	NSCLC	Upregulated	Serum	Elevated in serum of NSCLC patients. Part of a panel with high diagnostic accuracy (AUC 0.931 95% CI: 0.869–0.990).	–	–	(117)
	HIF1A-AS1	NSCLC						
	LINC00173	NSCLC			Serum levels higher in NSCLC patients. Diagnostic AUC of 0.809, improved when combined with CEA and Cyfra21-1.	62.96%	89.01%	(118)
	SChLAP1	NSCLC		Serum, Tissue	Upregulated in NSCLC serum, tissues, and cell lines. An excellent indicator for diagnosis and prognosis (AUC: 0.88 (95% CI: 0.7651 to 0.9948)).	–	–	(71)
	SNHG1	Lung cancer		Sputum	A sputum-based panel for lung cancer detection.	82.09%	89.23%	(119)
	H19							
	HOTAIR							
	SCARNA7	NSCLC		Pleural Fluid	A biomarker in pleural fluid for EGFR mutation status. Identified in pleural fluid as a biomarker for EGFR mutation status.	80.8%	67.1%	(121)
	MALAT1					79.2%	85.7%	
	NONHSAT017369					79.7%	63.6%	
	C5orf64	LUAD		Tissue	Expression correlated with M2 macrophage infiltration and immune checkpoint levels, serving as an indicator for tumor microenvironment remodeling.	–	–	(128)
Prognostic biomarker	TANlncSig	Lung cancer	–	Tissue	A neutrophil-specific lncRNA signature that independently stratifies patients into distinct survival groups, serving as a marker for myeloid cell infiltration and immunotherapy response.	–	–	(122)
	12-NETs-related Sig	NSCLC	–	Tissue	A 12-NETs-related lncRNA signature predicted shorter overall survival in high-risk patients.	–	–	(123)
	TILSig	NSCLC	–	Tissue	A signature that classified patients into immune-cold and immune-hot groups, where immune-hot patients exhibited prolonged survival and enhanced response to checkpoint inhibitors.	–	–	(124)
	LY6K-AS	LUAD	Upregulated	Tissue	Elevated expression correlated with poor LUAD survival and regulated mitotic progression.	–	–	(129)
	MIR155HG	LUAD		Tissue	High levels predicted better overall survival in LUAD and correlated with PD-1/PD-L1/CTLA-4 expression.	–	–	(130)
	LINC00669	LUAD		Tissue	The LINC00669-based signature combined with tumor stage achieved an AUC of 0.746 for prognosis, stratifying patients into risk subgroups.	–	–	(125)
	CTLA4LncSigs	LUAD	–	Tissue	A signature dynamically reflected treatment efficacy, with the risk score serving as an independent prognostic factor.	–	–	(126)

NSCLC, Non-small cell lung cancer; LUAD, Lung adenocarcinoma; AUC, Area under the curve; CI, Confidence interval; EGFR, Epidermal growth factor receptor; CEA, Carcinoembryonic antigen; Cyfra21-1, Cytokeratin 19 fragment; PD-1, Programmed cell death protein 1; PD-L1, Programmed death-ligand 1; CTLA-4, Cytotoxic T-lymphocyte-associated protein 4; TANlncSig, Tumor-associated neutrophil lncRNA signature; 12-NETs-related Sig, 12-Neutrophil extracellular traps-related signature; TILSig, Tumor-infiltrating lymphocyte signature; CTLA4LncSigs, CTLA-4-related lncRNA signatures.

### 4.2 lncRNAs as modulators of therapy resistance in lung cancer

**Figure 9:**
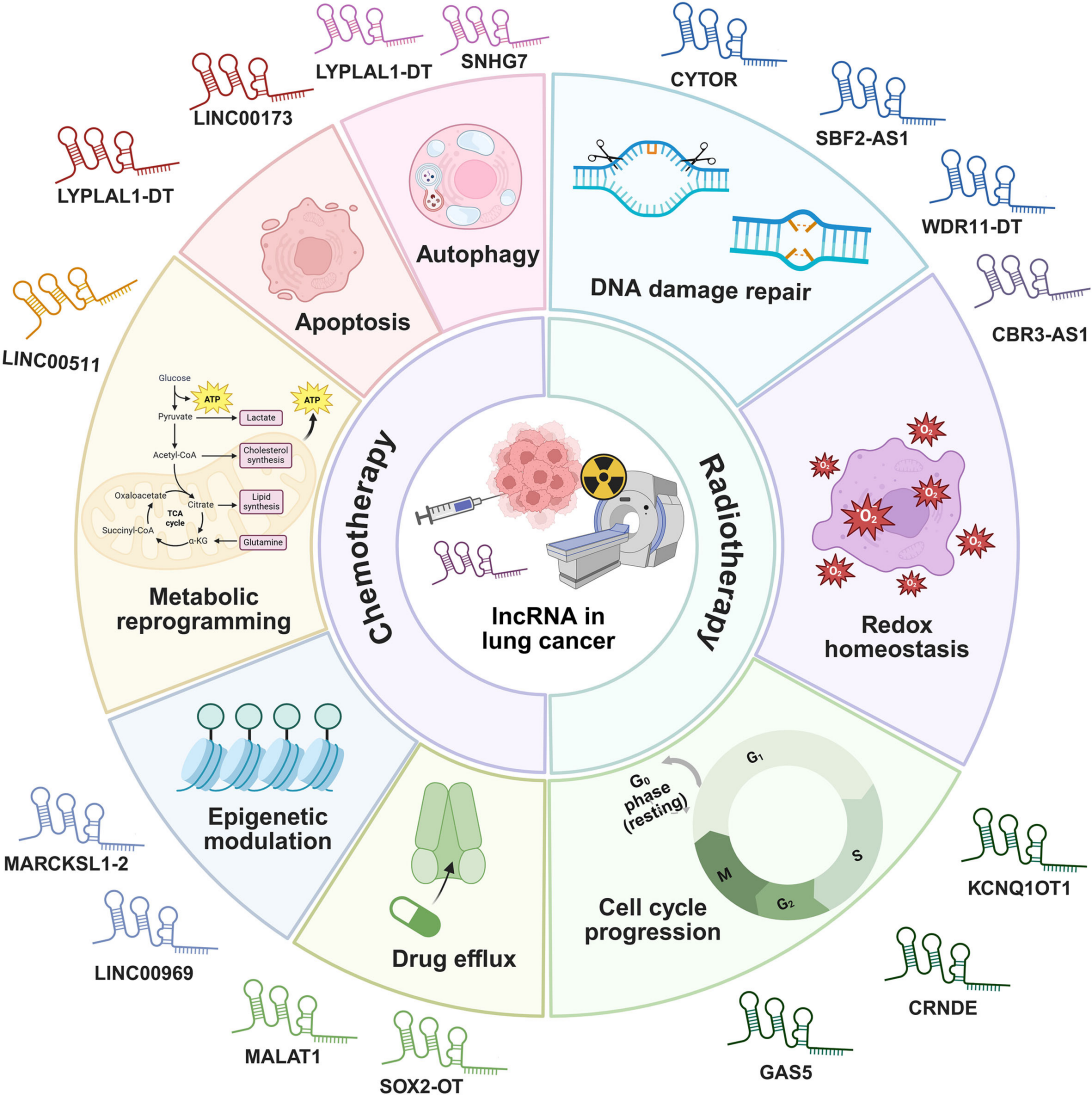
The mechanisms of lncRNAs regulating chemotherapy and radiotherapy responses in lung cancer.

While systemic therapies such as chemotherapy provide substantial benefits for patients of advanced lung cancer in both preoperative and postoperative settings, the high initial response rates are frequently followed by the development of therapeutic resistance and disease recurrence. This resistance remains a major obstacle to improving long-term survival, driven by complex molecular adaptations within the TME. Accumulating evidence indicates that lncRNAs function as critical regulators of these adaptive processes, mediating resistance through diverse mechanisms (**Figure 9**) (115, 116).

#### 4.2.1 lncRNAs in modulating chemotherapy resistance

Chemotherapy regimens, primarily involving agents such as cisplatin, etoposide, and paclitaxel, remain a cornerstone first-line treatment for advanced NSCLC and are also extensively used in SCLC (131). Despite initial efficacy, the majority of patients inevitably develop therapeutic resistance to these chemotherapeutic drugs. This chemotherapy resistance poses a paramount clinical challenge. Intrinsic and acquired resistance significantly curtail the long-term benefits of chemotherapy, highlighting an urgent need to decipher its molecular drivers and develop novel strategies to circumvent it (132). lncRNAs have emerged as pivotal regulators of these complex resistance mechanisms, offering new insights and therapeutic avenues (133). Chemotherapy resistance in lung cancer is orchestrated by lncRNAs through four molecular mechanisms including regulation of autophagy and apoptosis, metabolic reprogramming, epigenetic modulation, and control of drug efflux.

Regarding the regulation of autophagy and apoptosis, lncRNA LYPLAL1 divergent transcript (LYPLAL1-DT) is highly upregulated in multi-drug resistant SCLC and promotes resistance to cisplatin, etoposide, and paclitaxel by enhancing autophagy and suppressing apoptosis. It acts by sponging miR-204-5p to elevate BCL2 expression and facilitates the assembly of the beclin 1/PtdIns3K autophagy complex. Notably, combining venetoclax with hydroxychloroquine effectively counteracts this form of resistance in preclinical models (134). Another regulator of cell survival, LINC00173, plays a context-dependent role. It is downregulated in cisplatin-resistant LUAD, where its loss promotes resistance through the miR-1275/protein interacting with cyclin A1 (CCNA1)/zinc finger protein 36 like 2 (ZFP36L2) axis, increasing BCL2 mRNA stability and inhibiting apoptosis. Interestingly, it has also been reported as an oncogenic factor in SCLC and lung squamous cell carcinoma (LUSC), underscoring its subtype-specific functions (135).

lncRNAs also drive chemoresistance by reprogramming cellular metabolism. LINC00511 is downregulated by β-elemene, leading to inhibited expression of key glycolytic enzymes such as glucose transporter 1 (GLUT1), pyruvate kinase M2 (PKM2), and lactate dehydrogenase A (LDHA), as well as β-catenin, thereby reversing chemoresistance (136).

Beyond transcriptional and metabolic reprogramming, epigenetic modulation represents another critical layer of lncRNA-mediated chemoresistance. MARCKS like 1-2 (MARCKSL1-2) reverses docetaxel resistance by recruiting suppressor of zeste 12 homolog (SUZ12) to suppress histone deacetylase 1 (HDAC1) transcription via H3K27me3 modification, resulting in elevated miR-200b expression and restored drug sensitivity (137). Similarly, LINC00969 promotes gefitinib resistance through its interaction with EZH2 and methyltransferase-like 3 (METTL3), which leads to the epigenetic suppression of NLRP3. This process involves both H3K27me3 mediated transcriptional silencing and m^6^A-dependent mRNA decay, effectively inhibiting NLRP3 dependent pyroptosis and thereby facilitating drug resistance in lung cancer (138).

Furthermore, a key mechanism of resistance involves the lncRNA-mediated control of drug efflux. For instance, MALAT1 contributes to resistance by upregulating drug transporter genes, leading to increased efflux of chemotherapeutic agents and reduced intracellular drug accumulation (139). Other lncRNAs including SOX2-OT (48), SNHG7 (47), and MEG3 (69) have also been implicated in promoting chemotherapy resistance through above mechanisms.

#### 4.2.2 lncRNAs in modulating radiosensitivity

Radiotherapy is a pivotal and potentially curative modality for locally advanced lung cancer, both as a definitive treatment and in combination with chemotherapy or immunotherapy (140). However, radioresistance is a major impediment to achieving durable local control and survival. The biological basis of radioresistance involves a complex interplay of cellular processes designed to mitigate the cytotoxic effects of ionizing radiation. Key mechanisms include DNA damage repair, redox homeostasis, and cell cycle progression. Overcoming radioresistance is crucial for improving the therapeutic ratio of radiotherapy in lung cancer (141). Dysregulation of specific lncRNAs has been closely linked to these radioresistant phenotypes, positioning them as key therapeutic targets to enhance radiotherapeutic efficacy (133). Radiotherapy resistance in lung cancer is orchestrated by lncRNAs through above mechanisms (141).

DNA repair capacity is a key determinant of radiosensitivity. In this regard, lncRNA cytoskeleton regulator RNA (CYTOR) confers resistance by acting as a molecular sponge for miR-206, leading to upregulation of prothymosin α (PTMA), a protein that inhibits radiation-induced apoptosis and promotes DNA repair machinery (142). Similarly, lncRNA SBF2 antisense RNA 1 (SBF2-AS1) enhances radioresistance through the miR-302a/MBNL3 axis, where MBNL3 knockdown sensitizes tumor cells to DNA damage (143). In contrast, lncRNA WDR11 divergent transcript (WDR11-DT) is a radiation-induced lncRNA that is downregulated in NSCLC and associated with poor prognosis post-radiotherapy. It enhances radiosensitivity by promoting poly (ADP-ribose) polymerase 1 (PARP1) degradation via thyroid hormone receptor interactor 12 (TRIP12), impairing single-strand break repair, and binding heterogeneous nuclear ribonucleoprotein K (HNRNPK) to reduce the stability of homologous recombination genes (144).

Besides DNA repair capacity, the maintenance of redox homeostasis is another crucial determinant of radiosensitivity. This is well-illustrated by lncRNA CBR3-AS1, which is overexpressed in NSCLC and confers radioresistance by acting as a ceRNA for miR-409-3p, thereby upregulating superoxide dismutase 1 (SOD1). This alleviates radiation-induced oxidative stress and enhances DNA repair, whereas its knockdown increases γH2AX foci, ROS levels, and apoptosis (145). This model, supported by canonical validation, exemplifies the need to determine if such ceRNA activity represents the lncRNA’s primary function, while also highlighting that other potential mechanisms remain to be explored.

For autophagy-mediated survival, lncRNA KCNQ1 opposite strand/antisense transcript 1 (KCNQ1OT1) sequesters miR-372-3p to induce ATG 5/12-dependent autophagy, enabling tumor cell recovery after stereotactic radiotherapy (146). Additionally, lncRNA colorectal neoplasia differentially expressed (CRNDE) is upregulated in radioresistant LUAD cells and contributes to resistance by recruiting EZH2 to repress p21 transcription, facilitating G1/S transition and reducing apoptosis (147). Notably, tumor-suppressive lncRNAs like GAS5 can counteract resistance by inhibiting miR-135b or suppressing the miR-21/PTEN/AKT survival pathway to enhance radiosensitivity (148). Additionally, certain lncRNAs such as PCAT1 (40), AGAP2-AS1 (46), and CBR3-AS1 (54) are involved in immune escape by modulating immune cell activity. Their mechanism has been described previously.

Collectively, these findings position lncRNAs as master regulators of radiotherapy response through the mechanisms discussed above, offering promising targets for novel therapeutic strategies. **Table 3** summarizes the role of specific lncRNAs as key regulators of resistance to diverse treatment modalities.

**Table 3:** lncRNAs in lung cancer therapy resistance.

Clinical significance		lncRNAs	Cancer types	Regulation	Targets	Mechanisms	Ref.
Chemotherapy resistance	cDDP, VP-16 and PTX	LYPLAL1-DT	SCLC	Upregulated	miR-204-5p/BCL2	Promotes the expression of BCL2 by sponging miR-204-5p and is implicated in the assembly of the BECN1/PtdIns3K complex.	(134)
	Cisplatin	LINC00173	LUAD	Downregulated	miR-1275/PROCA1/ZFP36L2	Its loss promotes cisplatin resistance by impairing the adsorption of miR-1275 to suppress PROCA1/ZFP36L2-induced BCL2 degradation.	(135)
			LUSC and SCLC	Upregulated		It may act as an oncogene.	
		LINC00511	LUAD	Upregulated	GLUT1/PKM2/LDHA/β-catenin	Promotes chemoresistance by regulating glycolysis and the Wnt/β-catenin pathway.	(136)
		MALAT1	NSCLC	Upregulated	STAT3	Upregulates MRP1 and MDR1 transporters via STAT3 activation, facilitating cisplatin expulsion in NSCLC cells.	(139)
		MEG3	LCSC	Upregulated	miR-15a-5p/CCNE1	Promotes DDP chemoresistance via serving as a sponge of miR-15a-5p to mediate CCNE1 expression.	(69)
	Docetaxel	MARCKSL1-2	LUAD	Downregulated	SUZ12/HDAC1/miR-200b	Reverses docetaxel resistance by recruiting SUZ12 to suppress HDAC1 and elevate miR-200b.	(137)
	Gefitinib	LINC00969	Lung cancer	Upregulated	EZH2/METTL3/NLRP3	Promotes acquired gefitinib resistance by interacting with EZH2 and METTL3, epigenetically suppressing NLRP3 to inhibit NLRP3/caspase-1/GSDMD-related classical pyroptosis signaling pathways.	(138)
	EGFR-TKIs	SOX2-OT	NSCLC	Upregulated	miR-627-3p/Smads	Enhances the resistance to EGFR-TKIs through promoting Smads by sponging miR-627-3p to promote M2 polarization in macrophages.	(48)
	Docetaxel	SNHG7	LUAD	Upregulated	ATG5/12, PI3K/AKT	Enhances docetaxel resistance through stabilizing autophagy-related genes ATG5 and ATG12 and activating PI3K/AKT pathway to promote M2 polarization in macrophages.	(47)
Radiotherapy resistance		CYTOR	NSCLC	Upregulated	miR-206/PTMA	Enhances radioresistance by bounding to miR-206 and leading to upregulation of PTMA, which inhibits radiation-induced apoptosis and promotes DNA repair.	(142)
		WDR11-DT	NSCLC	Downregulated	PARP1/HNRNPK/HR genes	Enhances radiosensitivity by promoting PARP1 degradation and binding RNA-bind protein HNRNPK to suppress radiotherapy-triggered HR repair.	(144)
		SBF2-AS1	NSCLC	Upregulated	MBNL3	Enhances radioresistance through upregulation of MBNL3.	(143)
		KCNQ1OT1	LUAD	Upregulated	miR-372-3p/ATG5/12	Enhances radioresistance by sequestering miR-372-3p to induce ATG5/ATG12-dependent autophagy, enabling tumor cell recovery after radiotherapy.	(146)
		CRNDE	LUAD	Upregulated	EZH2/p21	Contributes to radioresistance by recruiting EZH2 to repress p21 transcription, facilitating cell cycle progression.	(147)
		PCAT1	NSCLC	Upregulated	cGAS/STING/SOX2	Enhances radioresistance by activating SOX2 to suppress cGAS/STING-mediated DNA sensing.	(40)
		AGAP2-AS1	Lung cancer	Upregulated	miR-296/NOTCH2	Enhances radiotherapy efficacy by sponging miR-296 to elevate NOTCH2, thereby promoting NK cell-mediated tumor clearance.	(46)
		CBR3-AS1	NSCLC	Upregulated	miR-409-3p/SOD1	Enhances radioresistance by functioning as an oncogene through the CBR3-AS1/miR-409-3p/SOD1 pathway	(145)
		GAS5	NSCLC	Downregulated	miR-135b, miR-21/PTEN/AKT	Enhances radiosensitivity by inhibiting miR-135b or suppressing the miR-21/PTEN/AKT survival pathway.	(148)

cDDP, cis-Diamminedichloroplatinum; VP-16, Etoposide; PTX, Paclitaxel; SCLC, Small cell lung cancer; LUAD, Lung adenocarcinoma; LUSC, Lung squamous cell carcinoma; NSCLC, Non-small cell lung cancer; miR, microRNA; BCL2, B-cell lymphoma 2; BECN1, Beclin 1; PROCA1, protein interacting with cyclin A1; ZFP36L2, Zinc finger protein 36, C3H1 type-like 2; GLUT1, Glucose transporter 1; PKM2, Pyruvate kinase M2; LDHA, Lactate dehydrogenase A; STAT3, Signal transducer and activator of transcription 3; CCNE1, Cyclin E1; PARP1, Poly(ADP-ribose) polymerase 1; SUZ12, Suppressor of zeste 12 homolog; HDAC1, Histone deacetylase 1; EZH2, Enhancer of zeste homolog 2; METTL3, Methyltransferase-like 3; NLRP3, NLR family pyrin domain containing 3; MRP1, Multidrug resistance-associated protein 1; MDR1, Multidrug resistance protein 1; EGFR-TKI, Epidermal growth factor receptor tyrosine kinase inhibitor; Smads, Mothers against decapentaplegic homologs; ATG5/12, Autophagy-related 5/12; PI3K/AKT, Phosphoinositide 3-kinase/Protein kinase B; PTMA, Prothymosin Alpha; HNRNPK, Heterogeneous nuclear ribonucleoprotein K; HR genes, Homologous recombination genes; MBNL3, Muscleblind like splicing regulator 3; cGAS/STING, Cyclic GMP-AMP synthase/Stimulator of interferon genes; SOX2, SRY (sex determining region Y)-box 2; NOTCH2, Neurogenic locus notch homolog protein 2; NK cell, Natural killer cell; SOD1, Superoxide Dismutase 1; CXCL1, C-X-C motif chemokine ligand 1; PTEN, Phosphatase and tensin homolog.

### 4.3 lncRNAs as therapeutic targets

The strategic disruption of oncogenic lncRNAs is increasingly recognized as a powerful approach to enhance the efficacy of standard cancer therapies (149). This is achieved through a multi-pronged strategy, from re-sensitizing tumors to conventional chemotherapy and augmenting immune checkpoint blockade, to employing advanced molecular tools like ASOs for targeted inhibition (**Table 4**). Ultimately, these strategies all share the same goal to circumvent resistance mechanisms and achieve superior antitumor outcomes through combination therapy (150).

**Table 4:** lncRNAs as therapeutic targets.

Clinical significance	lncRNAs	Cancer types	Regulation	Targets	Mechanisms	Ref.
Therapeutic target	LYPLAL1-DT	SCLC	Upregulated	miR-204-5p/BCL2/BECN1	Sequesters apoptosis through the LYPLAL1-DT/miR-204-5p/BCL2 axis and promotes autophagy by facilitating the assembly of the BECN1/PtdIns3K complex.	(134)
	LINC00973	Lung cancer	Upregulated	miR-216b/miR-150/CD55/CD59	Sponges miR-216b and miR-150 to upregulate CD55 and CD59 expression and suppress complement system activation to promote tumor immune evasion.	(39)
	LINC02418	NSCLC	Downregulated	Trim21/PD-L1	Targeting METTL3 to upregulate LINC02418 enhances Trim21-Mediated PD-L1 Ubiquitination and synergizes with anti-PD-1 therapy.	(72)
	MIR31HG	NSCLC	Upregulated	WDR5/MLL3/P300/GLI2	ASO-mediated knockdown of MIR31HG reduced tumor stemness and metastasis by recruiting the WDR5/MLL3/P300 complex to modulate the expression of GLI2.	(60)
	PKMYT1AR	NSCLC	Upregulated	miR-485-5p/PKMYT1/Wnt	ASOs targeting PKMYT1AR effectively suppressed tumor growth in preclinical models. By binding to PKMYT1AR, thereby disrupting the PKMYT1AR/miR-485-5p/PKMYT1 axis that normally promotes cancer stem cell maintenance via Wnt signaling activation.	(57)
	HIF1A-AS2	NSCLC	Upregulated	DHX9/MYC	Activates MYC by recruiting DHX9 on MYC promoter, consequently stimulating the transcription of MYC and its target genes.	(151)
	THOR	NSCLC	Upregulated	IGF2BP1/MYC	Enhances NSCLC cell proliferation, migration and invasion by directly binding IGF2BP1 protein in NSCLC cells.	(152)
	NLUCAT1	NSCLC	Upregulated	ALDH3A1/GPX2/GLRX/PDK4	Its knockout favors cisplatin-induced ROS-dependent apoptosis by upregulating ALDH3A1, GPX2, GLRX, PDK4 in LUAD cells.	(153)

NLUCAT1, lncRNA nuclear LUCAT1; SCLC, Small cell lung cancer; NSCLC, Non-small cell lung cancer; miR, microRNA; BCL2, B-cell lymphoma 2; BECN1, Beclin 1; PD-L1, Programmed death-ligand 1; WDR5, WD repeat domain 5; MLL3, Mixed lineage leukemia 3; P300, E1A binding protein p300; GLI2, GLI family zinc finger 2; PKMYT1, Protein Kinase, Membrane Associated Tyrosine/Threonine 1; DHX9, DEAH (Asp-Glu-Ala-His) box polypeptide 9; IGF2BP1, Insulin-like growth factor 2 mRNA binding protein 1; ALDH3A1, Aldehyde dehydrogenase 3 family, member A1; GPX2, Glutathione peroxidase 2; GLRX, Glutaredoxin; PDK4, pyruvate dehydrogenase kinase, isozyme 4; ASOs, Antisense oligonucleotides; Trim21, Tripartite motif containing 21; PD-1, Programmed cell death protein 1.

#### 4.3.1 Overcoming chemoresistance and improving immunotherapy by targeting lncRNAs

A primary focus has been on overcoming chemoresistance by targeting key oncogenic lncRNAs. For example, the lncRNA LYPLAL1 divergent transcript (LYPLAL1-DT) is significantly upregulated in multi-drug resistant SCLC. It contributes to therapy resistance through mechanisms involving apoptosis inhibition and pro-survival autophagy. Importantly, the synergistic combination of venetoclax and hydroxychloroquine effectively reversed this type of resistance in both cell line-derived and patient-derived xenograft models. This combination offers a promising therapeutic strategy to overcome lncRNA-mediated drug resistance (134).

Beyond their role in chemoresistance, lncRNAs represent compelling targets for modulating immunotherapy response. Efforts to overcome immune checkpoint blockade resistance have focused on lncRNAs that regulate PD-L1 expression. For instance, targeting the RNA-modifying enzyme METTL3 to upregulate the tumor-suppressive lncRNA LINC02418, which promotes PD-L1 degradation and synergizes with anti-PD-1 therapy (72). Additionally, targeting the downstream effectors CD55 and CD59 with neutralizing antibodies, which are upregulated via EGFR/Wnt signaling and LINC00973, activates the complement system and synergizes with anti-PD-1 therapy to achieve a robust antitumor response (39).

#### 4.3.2 Novel therapeutic strategies directly targeting lncRNAs

**Figure 10:**
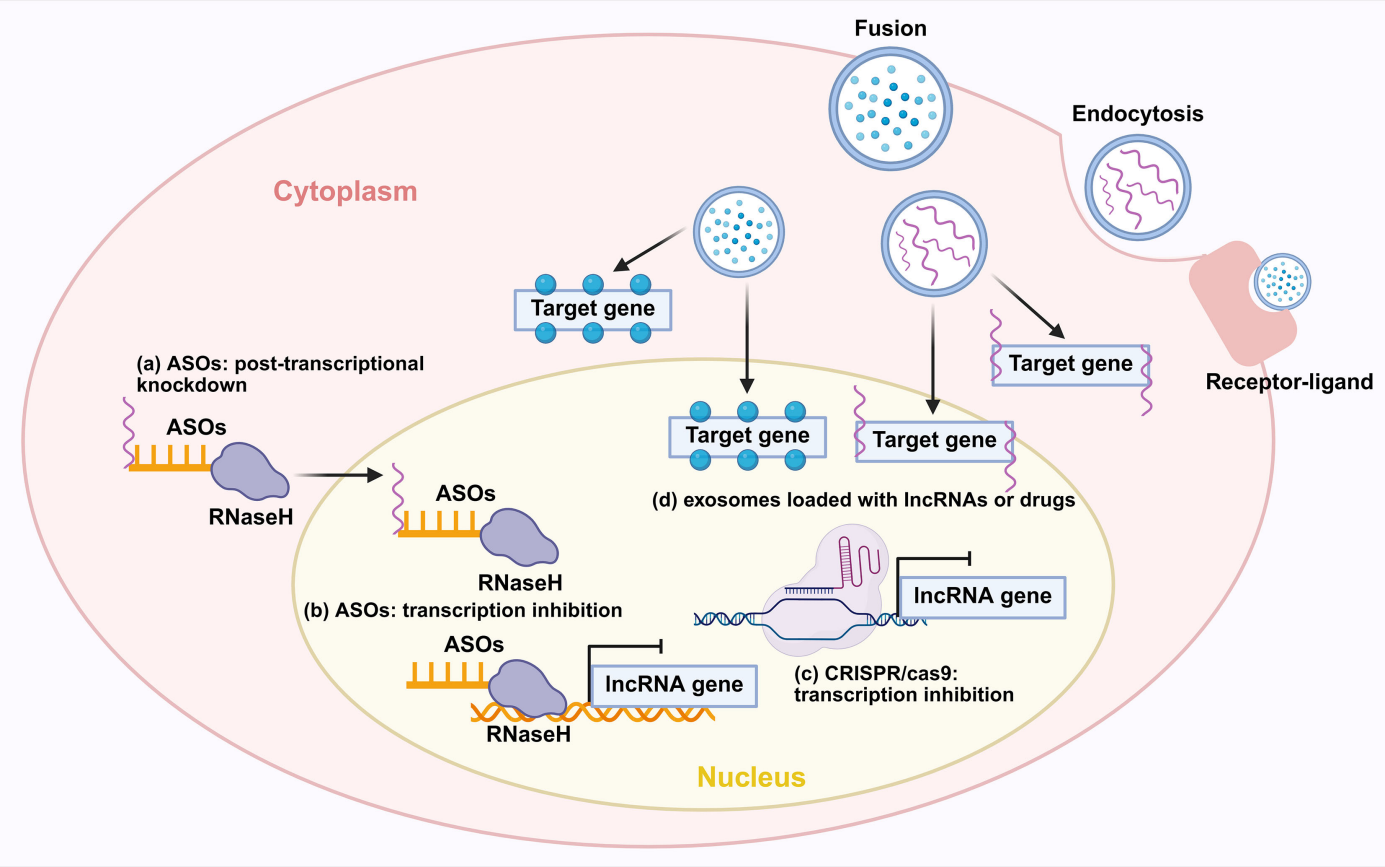
Novel therapeutic strategies for targeting lncRNAs. **(a)** ASOs exert effects through post-transcriptional knockdown via RNase H-mediated degradation of target lncRNAs. **(b)** ASOs inhibit transcription by interfering with the transcription of lncRNA genes. **(c)** CRISPR-Cas9 achieves transcriptional inhibition of lncRNA genes. **(d)** Exosomes loaded with lncRNAs or drugs can deliver therapeutic cargos. Exosomes can also be internalized into cells via receptor-ligand mediated endocytosis to modulate target genes. ASO, antisense oligonucleotide; RNaseH, Ribonuclease H.

The development of therapeutic strategies directly targeting lncRNAs is a promising avenue for lung cancer treatment, with major approaches including ASOs, the CRISPR/Cas9 system, and engineered exosomes (**Figure 10**).

ASOs represent a promising therapeutic strategy for targeting oncogenic lncRNAs. They function by binding to complementary RNA sequences through Watson-Crick base pairing, which subsequently recruits RNase H to degrade the target transcript. This mechanism offers high specificity and minimal off-target effects compared to conventional approaches. Furthermore, ongoing advancements are continuously improving the stability and delivery efficiency of ASO-based drugs (154). For instance, ASO-mediated knockdown of the oncogenic lncRNA MIR31HG reduces Treg-mediated immunosuppression and suppresses tumor growth (60). ASOs targeting the oncogenic lncRNA PKMYT1AR effectively suppressed tumor growth by modulating downstream signaling pathways (57). Similarly, ASO-mediated knockdown of ADPGK-AS1 reverses its pro-tumorigenic metabolic reprogramming of macrophages, shifting them from an M2 to a tumor-suppressive M1 phenotype and inhibiting lung tumor growth *in vivo* (41). Furthermore, by targeting the lncRNA HIF1A-AS2 with LNA GapmeR ASOs, researchers disrupted its double-positive feedback loop with MYC in KRAS-driven lung cancer. This intervention significantly inhibited tumor growth and enhanced the efficacy of both MYC inhibitors and cisplatin in patient-derived xenograft and transgenic mouse models (151). Currently, ASO-based therapeutics in lung cancer necessitate further exploration. However, with ongoing advancements in antisense oligonucleotide technology, research into their clinical application for lung cancer is anticipated to accelerate rapidly.

The CRISPR/Cas9 system offers a potent and definitive approach to target oncogenic lncRNAs in NSCLC. A key demonstration of this is the successful knockout (KO) of lncRNA THOR, which was achieved using a specific sgRNA in Cas9-expressing lung cancer cells. This genetic ablation disrupted the lnc-THOR/IGF2BP1 oncogenic axis, leading to the downregulation of key target mRNAs, and resulted in significantly suppressed tumor cell proliferation, migration, and increased apoptosis (152). In parallel, CRISPR/Cas9-mediated deletion of the hypoxia-regulated lncRNA nuclear LUCAT1 (NLUCAT1) robustly inhibited tumor aggressiveness. The knockout of NLUCAT1 not only attenuated cancer cell proliferation and invasion but also heightened oxidative stress and sensitized cells to cisplatin-induced apoptosis, underscoring its role in regulating the cellular response to chemotherapy (153). Collectively, these studies demonstrate that CRISPR/Cas9 provides a precise and irreversible means to inactivate oncogenic lncRNAs, presenting a promising gene-editing approach for lung cancer therapy. However, notwithstanding its precision, the clinical translation of CRISPR/Cas9 is tempered by concerns over potential off-target effects, which could lead to unintended genomic alterations and serious consequences. Addressing these limitations is crucial for the safe and effective application of this technology in future cancer treatments (155).

Beyond direct RNA targeting, exosomes, which are nanoscale extracellular vesicles secreted by various cells, have emerged as a novel and promising delivery system for therapeutic agents. They function as natural mediators of intercellular communication by shuttling functional molecules, including proteins, lipids, mRNAs, and non-coding RNAs such as lncRNAs and miRNAs, and play pivotal roles in tumor pathogenesis and progression (156). Critically, exosomes exhibit higher safety and superior bioavailability compared to traditional synthetic vectors, making them an attractive platform for targeted therapy (157). Consequently, engineering exosomes to deliver specific drugs or therapeutic lncRNAs represents a highly promising frontier for developing next-generation targeted therapies in lung cancer.

The clinical potential of lncRNA modulation will likely be realized through its integration into multi-targeted regimens (158). Combining lncRNA-directed agents with immune checkpoint blockade, cellular therapies, or conventional treatments offers a pathway to disrupt synergistic immunosuppressive mechanisms and prevent adaptive resistance. Key to this approach will be a deeper mechanistic understanding of lncRNA function in the TME and the development of biomarkers to identify responsive patient populations (39). A critical aspect of this understanding involves deciphering the complex ceRNA networks, where lncRNAs function as molecular sponges to sequester miRNAs, thereby modulating the expression of downstream target genes involved in tumor progression and therapy resistance (159). Ultimately lncRNA modulation will enable more durable and adaptive treatment responses.

## 5 Conclusion and future perspectives

This review has synthesized current knowledge on the critical roles of lncRNAs in remodeling the TME of lung cancer. We have discussed how lncRNAs regulate the function and polarization of key immune cells including T cells, TAMs, MDSCs, TANs, as well as stromal components such as CAFs and CSCs. Additionally, we highlighted their influence on immune checkpoint expression, cytokine signaling, and chemokine activity, collectively shaping an immunosuppressive landscape that promotes tumor progression and therapy resistance. Furthermore, we explored the clinical applicability of these findings, emphasizing the potential of lncRNAs as diagnostic and prognostic biomarkers, as well as novel therapeutic targets to enhance the therapy efficacy in lung cancer.

While considerable progress has been made, future research should prioritize the following directions: (i) It is crucial to address fundamental questions in the field, including whether the emergence of oncogenic lncRNAs is a stochastic process and how to establish definitive causal relationships between lncRNAs and tumor progression (160). (ii) Elucidating the mechanisms governing lncRNA biogenesis, subcellular localization, and degradation, and critically reevaluating the prevalent ceRNA mechanism in light of insufficient stoichiometric relationships between lncRNAs and miRNAs (161, 162). (iii) Overcoming clinical obstacles, such as developing non-invasive detection methods, differentiating malignant from benign nodules, and identifying standardized lncRNA panels with reliable reference values (163). (iv) Leveraging advanced ex-vivo models like patient-derived organoids and engineered immune co-cultures to better dissect lncRNA functions in a humanized context, and exploring lncRNA-immune crosstalk to uncover novel combinatorial immunotherapies. (v) Exploring the crosstalk between lncRNAs and the immune system may uncover novel combinatorial immunotherapies that overcome resistance to existing checkpoint inhibitors.

Sustained research efforts are critical for unraveling the functional complexities of lncRNAs and translating these discoveries into effective clinical applications. Successful clinical implementation of lncRNA-based approaches will create new opportunities for precision medicine and ultimately yield improved therapeutic benefits against cancer.
